# Knowledge, attitudes and practices regarding disaster preparedness among nursing students

**DOI:** 10.1590/1518-8345.8135.4855

**Published:** 2026-07-20

**Authors:** Josefina Delgado-Delgado, Eduardo Pérez-Castro, Cornelio Bueno-Brito

**Affiliations:** 1 Universidad Autónoma de Guerrero, Facultad de Enfermería Nº 2, Acapulco, Guerrero, Mexico.; 2 Universidad Autónoma de Guerrero, Chilpancingo, Guerrero, Mexico.

**Keywords:** Nursing, Nursing Students, Knowledge, Attitudes, Practices, Disaster Preparedness.

## Abstract

**Objective:**

to analyze the knowledge, attitudes, and practices regarding disaster preparedness among nursing students, in a municipality highly vulnerable to natural disasters, in relation to their preparation for such events.

**Method:**

a questionnaire was administered to 363 students, with a mean age of 21.54 years, most of whom were women (78.51%).

**Results:**

97.25% demonstrated adequate knowledge about disasters; however, only 66.67% were familiar with their college’s evacuation procedures. In terms of attitudes, 93.11% showed a positive disposition toward disaster preparedness, although only 26.72% had received continuing academic training on the subject. Regarding practices, only 20.39% reported adequate actions in emergency situations, and 19.01% considered that their university was prepared to face a natural disaster. A positive correlation was identified between the level of knowledge and practices, suggesting that a higher level of knowledge favors the application of effective measures in emergency contexts.

**Conclusion:**

although nursing students demonstrated favorable knowledge and attitudes, practices remain limited. These findings highlight the need to reinforce practical training in disaster management to strengthen their capacity to respond to emergencies.

## Highlights

**(1)** First study in Mexico on disaster preparedness among nursing students. **(2)** Adequate knowledge and favorable attitudes, but limited practices in disaster situations. **(3)** Higher level of knowledge is associated with better practices in emergencies and disasters. **(4)** Only 26.72% received continuing education in natural disaster management.

## Introduction

According to the World Health Organization, disasters are defined as “unforeseen situations that pose serious and immediate threats to public health or any public health situation that endangers the life or health of a significant number of people and requires immediate action”^1^. Disasters are often associated with serious physical, mental, environmental, and economic crises in the affected population^2^ ^-^ ^4^.

Mexico is a country vulnerable to natural disasters^5^. In recent decades, the country has experienced a wide variety of disasters associated with natural phenomena, mainly hurricanes, floods, and earthquakes^6^ ^-^ ^7^. In this context, Acapulco, a municipality located in the state of Guerrero, is significantly vulnerable due to its geographical location and constant exposure to these phenomena. On October 25, 2023, Acapulco suffered the devastating impact of Hurricane Otis, a category 5 storm that caused severe damage to infrastructure, the environment, and the local population^8^. Similarly, the floods caused by Tropical Storm John at the end of September 2024 highlighted the recurrence and magnitude of the disasters affecting the municipality. Therefore, disaster prevention is an essential priority to mitigate its negative impacts and protect the most vulnerable communities.

Given this scenario, several studies have explored the knowledge, attitudes, and practices (KAP) regarding disaster preparedness among university students in the health field who, as future professionals, must be adequately prepared to play a key role in managing and mitigating the impacts of these events^9^.

A multicenter study conducted with 2,546 medical students from nine countries in Latin America and the Caribbean aimed to describe their knowledge, perceptions, and practices regarding risks and disasters. The results revealed that 43.6% considered themselves prepared to help in the event of a natural disaster, while 91.2% considered that countries in the region were not adequately prepared to deal with this type of situation^10^. On the other hand, in a study conducted in Lebanon, the authors reported that medical students had moderate knowledge and a favorable attitude toward disaster medicine, which highlights the need for specific courses to strengthen their preparedness^11^. In Singapore, a study found that medical students had a limited level of knowledge about emergency preparedness and, in addition, most expressed interest in including disaster medicine in the curriculum, with the aim of improving their response capacity in emergencies^12^.

With regard to nursing students, several studies have highlighted the importance of strengthening their training in disaster management, given their fundamental role in primary care and immediate response to emergencies^13^ ^-^ ^14^. It has been identified that these students have a knowledge base, skills, and abilities that make them valuable and unique assets during disaster response efforts, which reinforces the need to improve their preparation to deal with this type of situation effectively^14^ ^-^ ^15^. A recent study in Northern Cyprus reported moderate levels of disaster preparedness among nursing students, and the authors highlighted the importance of implementing education based on the health belief model^16^. A survey of 206 students at a nursing college in Saudi Arabia revealed that 69% of participants had adequate knowledge, 72% had a positive attitude toward disaster preparedness, but 84% had inadequate practice^17^. Another study found that most students showed a favourable attitude toward disaster preparedness and perceived high competence in disaster nursing; however, they had insufficient knowledge and limited understanding of their role in emergency response^18^. A study in Turkey found that more than half of nursing students had a limited level of knowledge about disasters and highlighted weaknesses in the rehabilitation phase, which highlights the importance of strengthening practical training in emergency management^19^.

The training of nursing professionals is a fundamental pillar for preparedness and response to emergencies and disasters. However, the undergraduate nursing program at the *Facultad de Enfermería N. º 2* of the *Universidad Autónoma de Guerrero* (UAGro) does not include specific courses on disaster management. This absence from the curriculum is particularly relevant in a municipality such as Acapulco, one of the most vulnerable to these natural phenomena.

Currently, natural disasters are a growing concern due to their frequency and intensity, as well as their impact on public health, which is why it is necessary for healthcare professionals, especially nurses, to be prepared to respond in a timely manner in these critical situations^20^. The evaluation of their KAP in this context allows for the identification of positive aspects and areas for improvement in their training, which ensures that they have the necessary skills to act in a timely manner^14^ ^,^ ^20^. Adequate preparation of future nursing professionals not only enables a more efficient response to disasters but also contributes to mitigating their impacts and protecting vulnerable populations. Based on the above, the present study aimed to analyze the KAP of nursing students, in a municipality highly vulnerable to natural disasters, in relation to their preparation for such events.

## Method

### Study design

A cross-sectional observational study was conducted to explore nursing students’ KAPs in relation to disaster prevention, following the recommendations of the Strengthening the Reporting of Observational Studies in Epidemiology (STROBE) guide for observational studies^21^.

### Setting

The study was conducted at the *Facultad de Enfermería N.º 2* of the *Universidad Autónoma de Guerrero* (UAGro), located in the city and port of Acapulco, Guerrero, Mexico.

### Period

Data collection was conducted between November 25, 2024, and January 20, 2025.

### Population and selection criteria

The population consisted of students from the *Facultad de Enfermería N.º 2* of the UAGro, from the morning shift, in their second, third, and fourth years. A total of 460 students were invited to participate, of whom 363 completed the questionnaire, representing a response rate of 78.9%. Students who voluntarily agreed to participate and registered their response via an individual link sent by class representatives via institutional email were included. Incomplete questionnaires and duplicate records were excluded.

### Data collection tool

The instrument was adapted from a previously validated questionnaire used in another similar study^17^. Adaptations were made to the language and content to adjust it to the local context of nursing students at UAGro, maintaining the original structure of the KAP dimensions. Content validity was reviewed by two nursing professors. In addition to the expert review, a pilot study was conducted with a group of 12 students who were not part of the final sample. The students verified the clarity, comprehension, and relevance of the items, which allowed ambiguous expressions to be identified and corrected. This feedback ensured that the questionnaire was understandable to the target population. The final version of the instrument was structured in four sections: demographic characteristics of the students, which included variables such as age, gender, place of origin, and academic average; knowledge, assessed through 17 questions with “Yes” or “No” answer options, assigning 1 point for “Yes” and 0 for “No.” Knowledge was classified as unsatisfactory when the student obtained ≤ 10 correct answers (≤ 60%) and satisfactory when they exceeded this threshold; Attitudes, which consisted of 16 questions, were assessed on a Likert scale: 2 points for “Agree,” 1 point for “Neither agree nor disagree,” and 0 points for “Disagree.” Attitude was classified as positive when the score was ≥ 60% and negative when it was lower; practices, consisting of seven questions, which assessed student participation, training, and preparation in disaster management. Each response was scored with 1 point for “Yes” and 0 for “No.” Practice was considered satisfactory when the score was ≥ 60% and unsatisfactory when it was below this threshold.

Compared to the original questionnaire, additional questions were added in this adaptation and the distribution of items by dimension was modified, resulting in a greater number of items in the knowledge dimension (17 vs. 16), attitudes (16 vs. 11), and practices (7 vs. 3). These modifications were made with the purpose of expanding the practices section and adapting the instrument to the academic and professional reality of nursing students in Acapulco, without altering the logic of the KAP structure. In addition, two questions related to the perception of institutional preparedness for disasters were integrated, with the aim of enriching the evaluation of the studied context.

### Validation and reliability

The internal consistency of the questionnaire was assessed using the Kuder-Richardson 20 (KR-20) formula for sections with dichotomous responses. KR-20 reliability coefficients below 0.50 are considered low; those between 0.50 and 0.80 are considered moderate; and those above 0.80 are considered high^22^. Cronbach’s alpha was determined for the attitudes section, with values between 0.75 and 0.90 being considered acceptable.

### Data collection

The invitation was managed exclusively through class representatives, without involving faculty, in order to avoid bias in the teacher-student relationship. Initially, the link was sent by institutional email, along with an explanation of the study’s objectives and relevance. A second invitation was sent 15 days later to increase the response rate. Each student accessed the form through a unique registration on Google Forms, which ensured the individuality of the responses and avoided duplication.

### Data processing and analysis

The data were organized in a Microsoft Excel spreadsheet and subsequently analyzed using R statistical software. For qualitative variables (nominal and ordinal), relative frequencies and percentages were calculated, while continuous variables were expressed as mean and standard deviation (SD). Categorical variables were compared using Fisher’s exact test. Correlations between knowledge, attitude, and practice scores were calculated using Pearson’s correlation coefficient (r). For each correlation, a 95% confidence interval (95% CI) was obtained. A p-value < 0.05 was considered statistically significant.

### Ethical aspects

This study complied with the basic principles of bioethics and the Nursing Code of Ethics, as well as the provisions of the General Health Law Regulations regarding the ethical aspects of research involving human subjects. In addition, the international conventions contained in the Declaration of Helsinki were respected. The protocol was approved by the Research Ethics Committee of the *Facultad de Enfermería N.º 2* of UAGro, under protocol number PI-O1:10-11-2024.

## Results

The average age of the students who participated in the study was 21.54 years (SD = 3.12). Most participants were in the 21-23 age group (49.86%). Of the total, 78.51% (285) were women, while 21.49% (78) were men. Regarding their position in the course, 43.25% (157) of the students were in their fourth year, 28.93% (105) in their second year, and 27.82% (101) in their third year. Most respondents were single (95.04%), while 3.03% were married and 1.93% were in a stable relationship (Table 1). Regarding their current place of residence, 81.54% (296) lived with their family and 18.46% (67) lived away from their family. Most students were from Acapulco (79.06%), followed by regions such as Costa Chica (7.99%) and Costa Grande (7.99%). The students’ average grade for the previous semester was 9.20 (SD = 0.49) (Table 1).

**Table 1:** Sociodemographic and academic variables of nursing students. Acapulco, Guerrero, Mexico, 2024

Variable	Frequency (%)
Age (years)	
Average (SD*)	21.54 (3.12)
18-20	141 (38.84)
21-23	181 (49.86)
24 or older	41 (11.30)
Sex	
Male	78 (21.49)
Female	285 (78.51)
Position in the course	
Second	105 (28.93)
Third	101 (27.82)
Fourth	157 (43.25)
Marital status	
Single	345 (95.04)
Married	11 (3.03)
Common-law marriage	7 (1.93)
Who do you currently live with?	
With your family	296 (81.54)
Away from your family	67 (18.46)
Place of origin	
Acapulco	287 (79.06)
Costa Chica	29 (7.99)
Costa Grande	29 (7.99)
Montaña	10 (2.75)
Tierra Caliente	3 (0.83)
North	1 (0.28)
Previous semester average		
Mean (SD*)	9.20 (0.49)

*SD = Standard deviation

The internal consistency of the knowledge section, assessed using the KR-20 coefficient, was 0.61, indicating moderate reliability of the scale. 

Among the students interviewed, 97.25% (353) indicated that they were familiar with the concept of disaster, and 98.62% (358) recognized that the city where they lived could suffer a disaster at some point. A total of 92.29% (335) of students indicated that they were aware of the disasters most likely to affect their city, and 97.80% (355) considered that a disaster could affect their college facilities. However, only 66.67% (242) had received information in their academic training about the importance of organization in dealing with disasters. Of the total, 91.74% (333) of students said they knew what to do in the event of an earthquake; however, only 65.01% (236) reported being familiar with emergency evacuation procedures at their college. In addition, 88.43% (321) of students indicated that they knew what actions to take in the event of a hurricane or tropical storm, but only 59.23% (215) stated that they knew the safest place to be during a flood. The level of knowledge about disaster preparedness among nursing students was predominantly adequate.

According to the results, 96.97% (352) of participants demonstrated an adequate level of knowledge, while only 3.03% (11) demonstrated an inadequate level. The average score was 14.54 (SD=2.07) (Table 2).

**Table 2:** Nursing students’ knowledge of disaster preparedness. Acapulco, Guerrero, Mexico, 2024

Questions about knowledge	Correct (%)	Incorrect (%)
1. Have you heard of the concept of disaster?	353 (97.25)	10 (2.75)
2. During your college education, did they talk to you about the importance of organization in dealing with disasters?	242 (66.67)	121 (33.33)
3. Disasters are serious disruptions to the functioning of a community that exceed its capacity to cope with them using its own resources.	320 (88.15)	43 (11.85)
4. Are both natural and man-made disasters possible?	305 (84.02)	58 (15.98)
5. Could the city where you currently live suffer a disaster someday?	358 (98.62)	5 (1.38)
6. Do you know what disasters are most likely to affect the city where you live?	335 (92.29)	28 (7.71)
7. Do you believe that a disaster could, at some point, affect your nursing school’s facilities?	355 (97.80)	8 (2.20)
8. Disaster planning is the process of preparing for catastrophes by deciding what should be done and how it should be done.	342 (94.21)	21 (5.79)
9. Do you know why it is important to identify and address nearby threats that could cause a disaster at your college or in your community?	333 (91.74)	30 (8.26)
10. Are you familiar with the emergency evacuation procedures at your college in case of a disaster?	236 (65.01)	127 (34.99)
11. Do you know the exact locations of the emergency exit doors at your college?	245 (67.49)	118 (32.51)
12. Do you know what to do in the event of an earthquake?	333 (91.74)	30 (8.26)
13. Do you know where the safest place is during a flood?	215 (59.23)	148 (40.77)
14. Do you know what actions are necessary to prepare for the arrival of a hurricane or tropical storm?	321 (88.43)	42 (11.57)
15. Do you know if disaster response in your city involves the participation of health personnel (such as physicians and nurses) and non-health personnel (such as firefighters, police officers, or volunteers)?	291 (80.17)	72 (19.83)
16. Do you know what simulations are?	358 (98.62)	5 (1.38)
17. Do you understand the role you should play during a simulation?	339 (93.39)	24 (6.61)
Level of knowledge	Appropriate	352 (96.97)
	Inappropriate	11 (3.03)
Average score for level of knowledge	Mean (SD*)	14.54 (2.07)

*SD = Standard deviation

The internal consistency of the attitudes section, assessed using Cronbach’s alpha, was 0.85, indicating high reliability for this section.

Of the total, 91.46% (332) of students considered it essential to have a disaster response plan and to identify and reduce potential risks. In addition, 90.63% (329) stated that disaster preparedness training is essential for the entire university community, while 85.95% (312) said that this training should be an integral part of the professional training of nursing students. A total of 85.67% (311) believed that it is important for the university to organize disaster simulations on a regular basis, and 85.40% (310) reported that these exercises would improve the preparedness of the university community. Among respondents, 66.39% (241) considered that disaster preparedness should be part of the mandatory curriculum of all health-related educational programs. On the other hand, 88.15% (320) reported that nursing students should be trained not only in health care but also in emotional and psychological management during disasters. Of the total, 85.67% (311) pointed out that it is important for students to actively participate in simulations, and 80.72% (293) considered that these exercises should include scenarios of natural disasters and emergencies caused by accidents or violence. In addition, 90.36% (328) agreed that university managers should be trained to lead in disaster situations, and 88.98% (323) recognized that this responsibility includes all members of the university community (Table 3).

The level of attitude toward disaster preparedness and response showed that 93.11% (338) of students had a positive attitude, while 6.89% (25) had a negative attitude. The average attitude score was 26.79 (SD = 4.33), indicating that, in general, students demonstrate a favorable disposition toward disaster preparedness.

**Table 3:** Nursing students’ attitudes toward disaster preparedness. Acapulco, Guerrero, Mexico, 2024

Questions about attitudes	Agree (%)	Neither agree nor disagree (%)	Disagree (%)
1. I do not consider it necessary to receive information about disaster preparedness and planning.	63 (17.36)	255 (70.25)	45 (12.40)
2. Do you believe that university managers should be properly trained to lead and deal with disaster situations?	328 (90.36)	22 (6.06)	13 (3.58)
3. Do you consider disaster preparedness and planning to be a responsibility that includes all members of the university community (managers, students, and administrative staff)?	323 (88.98)	26 (7.16)	14 (3.86)
4. I consider it important to identify and reduce potential risks that could lead to disasters.	332 (91.46)	21 (5.79)	10 (2.75)
5. I consider it essential to have a disaster response plan in place.	332 (91.46)	22 (6.06)	9 (2.48)
6. It is important that disaster preparedness plans are reviewed and updated periodically to ensure their effectiveness.	327 (90.08)	26 (7.16)	10 (2.75)
7. To what extent do you agree with the following statement: “I believe that the probability of a disaster occurring at my university is low”?	52 (14.33)	143 (39.39)	168 (46.28)
8. To what extent do you agree with the following statement: “It is important for the university to organize regular disaster drills to improve community preparedness”?	311 (85.67)	47 (12.95)	5 (1.38)
9. I believe that disaster preparedness training is essential for all university staff.	329 (90.63)	29 (7.99)	5 (1.38)
10. Do you consider it essential that disaster preparedness training be an integral part of the professional training of nursing students?	312 (85.95)	42 (11.57)	9 (2.48)
11. Do you think that conducting regular disaster simulations at your college would help improve the university community’s preparedness?	310 (85.40)	48 (13.22)	5 (1.38)
12. Nursing students should be trained not only in health care, but also in emotional and psychological management during disasters.	320 (88.15)	37 (10.19)	6 (2.48)
13. To what extent do you agree with the following statement: “Disaster simulations should include both natural disaster scenarios and emergencies caused by accidents or violent situations”?	293 (80.72)	52 (14.33)	18 (4.96)
14. To what extent do you agree with the following statement: “It is important for nursing students to actively participate in disaster simulations”?	311 (85.67)	46 (12.67)	6 (1.65)
15. Do you consider disaster simulations to be a good opportunity to improve collaboration between different areas of the university?	298 (82.09)	57 (15.70)	8 (2.20)
16. Disaster preparedness should be part of the mandatory curriculum of all health-related educational programs.	241 (66.39)	99 (27.27)	23 (6.34)
Attitude level	Positive	338 (93.11)	
	Negative	25 (6.89)	
Average attitude score	Mean(SD*)	26.79 (4.33)	

*SD = Standard deviation

The internal consistency of the practices section, assessed using the KR-20 coefficient, was 0.75, indicating moderate reliability of the scale.

Table 4 shows some of the results related to student practices. Only 26.72% (97) of students received continuing academic training in disaster management as part of their study program, while 73.28% (266) did not. Of the total, 66.12% (240) of respondents mentioned that disaster simulations (earthquakes, floods, fires, hurricanes) are carried out at their college, although 33.88% (163) indicated that they were not. Only 24.79% (90) attended training sessions or workshops organized by the university on disaster preparedness. A total of 22.87% (83) of students actively participated in activities related to disaster prevention in their university community. Of the total, 50.69% (184) of students reported being familiar with natural disaster response protocols in the locations where they perform their clinical practices or social service. Only 37.47% (136) received training to perform first aid in the event of a natural disaster.

Only 20.39% (74) of students demonstrated adequate practices, while 79.61% (289) demonstrated inadequate practices. The average score for practices was 2.85 (SD = 2.07), indicating a low level of application of practical measures in emergency situations.

**Table 4:** Nursing students’ practices regarding disaster preparedness. Acapulco, Guerrero, Mexico, 2024

Questions about practices	Yes (%)	No (%)
1. Do you receive ongoing academic training in disaster management as part of your study program?	97 (26.72)	266 (73.28)
2. Does your college conduct disaster simulations, such as earthquakes, floods, fires, or hurricanes?	240 (66.12)	123 (33.88)
3. Have you participated in training or workshops on disaster preparedness organized by your university?	90 (24.79)	273 (75.21)
4. Do you actively participate in disaster prevention activities in your university community?	83 (22.87)	280 (77.13)
5. At the place where you carry out your clinical, professional, or social service practices, are you familiar with the protocols for responding to natural disasters?	184 (50.69)	179 (49.31)
6. In the event of a natural disaster, have you received training in first aid?	136 (37.47)	227 (62.53)
7. In a disaster situation, would you feel capable of coordinating or supporting emergency work at your college/institution or in your city?	207 (57.02)	156 (42.98)
Level of practices	Adequate	74 (20.39)
	Inadequate	289 (79.61)
Average score for practices	Mean (SD*)	2.85 (2.07)

*SD = Standard deviation

As for students’ perceptions of institutional preparedness for disasters, only 20.66% (75) of students considered that the drills carried out at their university were sufficient to adequately prepare them for a real disaster, while 48.21% (175) had a neutral perception and 31.13% (113) believed that they were insufficient. On the other hand, only 19.01% (69) of students perceived that their university was well prepared to face a natural disaster, while 53.44% (194) expressed a neutral perception and 27.55% (100) considered that it was not.

No significant differences in knowledge and attitude were found according to age, grade, gender, residence, or academic performance (p > 0.05). However, fourth-year students performed more appropriate practices compared to those in lower grades (p = 0.03), while age, gender, residence, and academic performance did not influence practices (p > 0.05) (Table 5).

**Table 5:** Distribution of knowledge, attitude, and practices according to students’ sociodemographic characteristics. Acapulco, Guerrero, Mexico, 2024

Age (years)				
	18-20 n (%)	21-23 n (%)	24 or more n (%)	p*
Knowledge				
Inadequate	3 (0.83)	8 (2.20)	0 (0.00)	0.36
Adequate	138 (38.02)	173 (47.66)	41 (11.29)	
Attitude				
Positive	132 (36.36)	167 (46.01)	39 (10.74)	0.83
Negative	9 (2.48)	14 (3.86)	2 (0.65)	
Practice				
Adequate	118 (32.51)	141 (38.84)	30 (8.26)	0.23
Inadequate	23 (6.34)	40 (11.02)	11 (3.03)	
**Position in the course (year)**				
	**Second** **n (%)**	**Third** **n (%)**	**Fourth** **n (%)**	
Knowledge				
Inadequate	2 (0.55)	2 (0.55)	7 (1.93)	0.50
Adequate	103 (28.37)	99 (27.77)	150 (41.32)	
Attitude				
Positive	99 (27.27)	96 (26.45)	143 (39.39)	0.46
Negative	6 (1.65)	5 (1.38)	14 (3.86)	
Practice				
Adequate	13 (3.58)	21 (5.79)	40 (11.02)	0.03
Inadequate	92 (25.34)	80 (22.04)	117 (32.23)	
**Sex**				
	**Male** **n (%)**		**Female**	
Knowledge				
Inadequate	3 (0.83)		8 (2.20)	0.70
Adequate	75 (20.66)		277 (76.31)	
Attitude				
Positive	69 (19.01)		269 (74.10)	0.07
Negative	9 (2.48)		16 (4.41)	
Practice				
Adequate	22 (6.06)		52 (14.33)	0.06
Inadequate	56 (15.43)		233 (64.19)	
	**With your Family** **n (%)**		**Far from your Family** **n (%)**	
Knowledge				
Inadequate	11 (3.03)		0 (0.00)	0.22
Adequate	285 (78.51)		67 (18.46)	
Attitude				
Positive	274 (75.48)		64 (17.63)	0.60
Negative	22 (6.06)		3 (0.83)	
Practice				
Adequate	56 (15.43)		18 (4.96)	0.17
Inadequate	240 (66.12)		49 (13.50)	
**Previous semester average**				
	**≥8.5** **n (%)**		**<8.5** **n (%)**	
Knowledge				
Inadequate	10 (2.75)		1 (0.28)	1.00
Adequate	320 (88.15)		32 (8.82)	
Attitude				
Positive	308 (84.85)		30 (8.26)	0.48
Negative	22 (6.06)		3 (0.83)	
Practice					
Adequate	68 (18.73)		6 (1.65)	1.00
Inadequate	262 (72.18)		27 (7.44)	

*p = p-value of Fisher’s exact test

The total scores for knowledge, attitudes, and practices were analyzed using Pearson’s correlation coefficient. A moderate positive correlation was observed between knowledge and practices (r = 0.35; 95% CI: 0.25, 0.43), suggesting that a higher level of knowledge is associated with better practices. The relationship between knowledge and attitude was weak (r = 0.07; 95% CI: -0.03, 0.17), while the relationship between attitude and practice was slightly negative (r = -0.07; 95% CI: -0.17, 0.02), with no statistical significance in either case.

## Discussion 

The constant increase, complexity, and severity of disasters worldwide, and particularly in Mexico, highlight the importance of having trained nursing staff who can ensure safe and timely care in emergency situations^23^. This study evaluated, for the first time in southern Mexico, the level of disaster preparedness among nursing students at the *Facultad de Enfermería N.º 2* de Acapulco, Guerrero, an area highly vulnerable to natural phenomena.

The results show that the vast majority of students (96.97%) had an adequate level of knowledge about disaster preparedness, a value higher than that reported in Cyprus and Saudi Arabia^16^ ^-^ ^17^ and comparable to that of multicenter studies in Latin America and the Caribbean. This suggests that, in the Mexican context, nursing students have a solid theoretical foundation, probably related to study plans and exposure to catastrophic events in the region.

With regard to attitudes, 93.11% of participants expressed a positive attitude toward disaster preparedness and response, which is consistent with international findings that point to favourable attitudes despite limitations in technical knowledge or practical experience^11^ ^-^ ^12^ ^,^ ^18^. Studies in Turkey have shown that specific courses in disaster nursing improve students’ awareness and self-efficacy, elements closely linked to a proactive attitude toward emergencies^24^.

However, appropriate practices were limited: only 20.39% of students took effective action. This finding reflects a critical gap between knowledge and action, a pattern described in other contexts in which, despite adequate levels of knowledge and positive attitudes, students lack sufficient practical experience^16^ ^-^ ^19^. Progressive clinical exposure seems to have an influence, since fourth-year students performed significantly more appropriate practices than those in lower years (p = 0.03).

The correlation analysis showed a moderate relationship between knowledge and practice, while the relationship between knowledge and attitude, as well as between attitude and practice, was weak and insignificant. This indicates that theoretical training and motivation do not automatically translate into effective practices, which highlights the need for strategies that integrate simulations, workshops, and practical experiences to strengthen the effective preparation of students.

The study provides relevant evidence on the discrepancies between the KAPs of nursing students in southern Mexico; it complements the international literature and highlights the need to incorporate systematic practical skills into the curriculum. Currently, undergraduate nursing programs address disaster-related topics in isolated subjects, such as public health and first aid, but there is no comprehensive course that combines prevention, mitigation, and response to emergencies. Given the increasing frequency and intensity of disasters linked to climate change, it is a priority to strengthen practical training and collaboration with civil protection and health agencies.

It is recommended that nursing institutions implement continuing education programs and practical workshops, especially in regions with a high probability of disasters^23^ ^-^ ^26^. Future research could incorporate qualitative methods to explore students’ perceptions, barriers, and needs, which would allow for the design of more effective interventions^15^ ^,^ ^26^.

Among the limitations, we highlight the cross-sectional design, which prevents establishing causal relationships, and the inclusion of students from a single college, which may limit the generalization of the results. We suggest replicating the study with a more robust design and greater geographic coverage in order to strengthen the conclusions.

## Conclusion

Nursing students at the *Facultad de Enfermería N.º 2* de Acapulco demonstrated an adequate level of knowledge and a positive attitude toward disaster preparedness. However, appropriate practices were limited, highlighting a gap between theory and action. Fourth-year students demonstrated better practices than those in lower years, suggesting the importance of progressive training and clinical experience. It is necessary to incorporate these topics into the curriculum and strengthen practical training, as most students were unfamiliar with disaster response protocols and first aid management.

## Data Availability

Datasets related to this article will be available upon request to the corresponding author.
