# Supplementary material for: Knowledge, attitudes and practices regarding disaster preparedness among nursing students
Source: Rev Lat Am Enfermagem. 2026 Jul 20;34:e4855. doi: 10.1590/1518-8345.8135.4855 (PMC13387773; doi:10.1590/1518-8345.8135.4855)
Supplement: Translation

# Translation: Conhecimentos, atitudes e práticas sobre preparação para desastres em estudantes de enfermagem

**DOI:** 10.1590/1518-8345.8135.4856

**Authors:** Josefina Delgado-Delgado, Eduardo Pérez-Castro, Cornelio Bueno-Brito

**Affiliations:** 1 Universidad Autónoma de Guerrero, Facultad de Enfermería Nº 2, Acapulco, Guerrero, Mexico.; 2 Universidad Autónoma de Guerrero, Chilpancingo, Guerrero, Mexico.

**Keywords:** Enfermagem, Estudantes de Enfermagem, Conhecimento, Atitudes, Práticas, Preparação em Desastres

## Abstract

**Objetivo:**

analisar os conhecimentos, atitudes e práticas sobre a preparação para desastres em estudantes de enfermagem de um município altamente vulnerável a desastres naturais, em relação à sua preparação diante desses eventos.

**Método:**

foi aplicado um questionário a 363 estudantes, com idade média de 21,54 anos, em sua maioria mulheres (78,51%).

**Resultados:**

97,25% apresentaram conhecimentos adequados sobre desastres, no entanto, apenas 66,67% conheciam os procedimentos de evacuação de sua faculdade. Quanto às atitudes, 93,11% mostraram disposição positiva em relação à preparação para desastres, embora apenas 26,72% tivessem recebido formação acadêmica continuada sobre o tema. Em relação às práticas, somente 20,39% relataram ações adequadas em situações de emergência e 19,01% consideraram que sua universidade estava preparada para enfrentar um desastre natural. Identificou-se correlação positiva entre o nível de conhecimento e as práticas, o que sugere que maior nível de conhecimento favorece a aplicação de medidas efetivas em contextos de emergência.

**Conclusão:**

embora os estudantes de enfermagem tenham apresentado conhecimentos e atitudes favoráveis, as práticas continuam sendo limitadas. Esses achados evidenciam a necessidade de reforçar a formação prática em gestão de desastres para fortalecer sua capacidade de resposta diante de emergências.

## Destaques

**(1)** Primeiro estudo no México sobre preparação para desastres em estudantes de enfermagem. **(2)** Conhecimentos adequados e atitudes favoráveis, porém práticas limitadas em situações de desastres. **(3)** Maior nível de conhecimento associa-se a melhores práticas diante de emergências e desastres. **(4)** Apenas 26,72% receberam formação continuada em gestão de desastres naturais.

## Introdução 

De acordo com a Organização Mundial da Saúde, os desastres são definidos como “situações imprevistas que representam sérias e imediatas ameaças para a saúde pública ou qualquer situação de saúde pública que coloca em perigo a vida ou a saúde de uma quantidade significativa de pessoas e exige ação imediata”^1^. Os desastres costumam estar associados a graves crises físicas, mentais, ambientais e econômicas na população afetada^2^ ^-^ ^4^.

O México é um país vulnerável aos desastres naturais^5^. Nas últimas décadas, o país tem experimentado uma grande variedade de desastres associados a fenômenos naturais, principalmente furacões, inundações e sismos^6^ ^-^ ^7^. Nesse contexto, Acapulco, um município situado no estado de Guerrero, apresenta vulnerabilidade significativa devido à sua localização geográfica e à constante exposição a esses fenômenos. Em 25 de outubro de 2023, Acapulco sofreu o devastador impacto do furacão Otis, de categoria 5, que ocasionou graves danos à infraestrutura, ao meio ambiente e à população local^8^. Do mesmo modo, as inundações ocasionadas pela tempestade tropical John, no final de setembro de 2024, evidenciaram a recorrência e a magnitude dos desastres que afetam o município. Por isso, a prevenção de desastres constitui uma prioridade essencial para mitigar seus impactos negativos e proteger as comunidades mais vulneráveis.

Diante desse panorama, diversos estudos têm explorado os conhecimentos, atitudes e práticas (CAP) sobre a preparação para desastres em estudantes universitários da área da saúde que, como futuros profissionais, devem estar adequadamente preparados para desempenhar um papel fundamental na gestão e mitigação dos impactos desses eventos^9^.

Um estudo multicêntrico realizado com 2.546 estudantes de medicina de nove países da América Latina e do Caribe teve como objetivo descrever os conhecimentos, percepções e práticas sobre riscos e desastres. Os resultados revelaram que 43,6% se consideraram preparados para ajudar em caso de um desastre natural, enquanto 91,2% consideraram que os países da região não estavam adequadamente preparados para enfrentar esse tipo de situação^10^. Por outro lado, em um estudo realizado no Líbano, os autores relataram que os estudantes de medicina apresentaram conhecimento moderado e atitude favorável em relação à medicina de desastres, o que ressalta a necessidade de cursos específicos para fortalecer sua preparação^11^. Em Cingapura, um estudo constatou que os estudantes de medicina apresentaram nível limitado de conhecimentos sobre preparação para emergências e, além disso, a maioria manifestou interesse em incluir a medicina de desastres no plano de estudos, com o objetivo de melhorar sua capacidade de resposta diante de emergências^12^.

No que diz respeito aos estudantes de enfermagem, diversos estudos têm destacado a importância de fortalecer sua formação em gestão de desastres, dado seu papel fundamental na atenção primária e na resposta imediata às emergências^13^ ^-^ ^14^. Identificou-se que esses estudantes possuem uma base de conhecimentos, habilidades e capacidades que os converte em ativos valiosos e únicos durante os esforços de resposta a desastres, o que reforça a necessidade de melhorar sua preparação para enfrentar esse tipo de situação de forma eficaz^14^ ^-^ ^15^. Um estudo recente no norte de Chipre relatou níveis moderados de preparação para desastres em estudantes de enfermagem, e os autores destacaram a importância de implementar uma educação baseada no modelo de crenças em saúde^16^. Uma investigação realizada com 206 estudantes de uma faculdade de enfermagem na Arábia Saudita revelou que 69% dos participantes tinham conhecimentos adequados, 72% apresentavam atitude positiva em relação à preparação para desastres, no entanto, 84% apresentavam prática inadequada^17^. Outro estudo identificou que a maioria dos estudantes mostrou atitude favorável em relação à preparação para desastres e percebeu alta competência em enfermagem de desastres, entretanto, apresentou conhecimentos insuficientes e compreensão limitada de seu papel na resposta às emergências^18^. Um estudo na Turquia constatou que mais da metade dos estudantes de enfermagem apresentava nível limitado de conhecimentos sobre desastres e destacou fragilidades na fase de reabilitação, o que ressalta a importância de fortalecer a formação prática em gestão de emergências^19^.

A formação de profissionais de enfermagem constitui um pilar fundamental para a preparação e a resposta diante de emergências e desastres. No entanto, o plano de estudos da graduação em Enfermagem da *Facultad de Enfermería N. º 2* da *Universidad Autónoma de Guerrero* (UAGro) não contempla disciplinas específicas sobre gestão de desastres. Essa ausência curricular adquire especial relevância em um município como Acapulco, um dos mais vulneráveis a esses fenômenos naturais.

Atualmente, os desastres naturais são uma preocupação crescente devido à sua frequência e intensidade, bem como ao seu impacto na saúde pública, razão pela qual é necessário que os profissionais de saúde, especialmente enfermeiras e enfermeiros, estejam preparados para responder de maneira oportuna nessas situações críticas^20^. A avaliação de seus CAP neste âmbito permite identificar aspectos positivos e áreas de melhoria em sua formação, o que garante que disponham das competências necessárias para atuar de forma oportuna^14^ ^,^ ^20^. A adequada preparação dos futuros profissionais de enfermagem não apenas possibilita uma resposta mais eficiente diante dos desastres, como também contribui para a mitigação de seus impactos e para a proteção das populações vulneráveis. A partir do exposto, o presente estudo teve como objetivo analisar os CAP dos estudantes de Enfermagem de um município altamente vulnerável a desastres naturais, em relação à sua preparação diante desses eventos.

## Método

### Desenho do estudo

Realizou-se um estudo observacional transversal com o objetivo de explorar os CAP dos estudantes de enfermagem em relação à prevenção de desastres, seguindo as recomendações do guia *Strengthening the Reporting of Observational Studies in Epidemiology* (STROBE) para estudos observacionais^21^.

### Local

O estudo foi realizado na *Facultad de Enfermería N.º 2* da *Universidad Autónoma de Guerrero* (UAGro), localizada na cidade e porto de Acapulco, Guerrero, México.

### Período

A coleta de dados foi realizada entre 25 de novembro de 2024 e 20 de janeiro de 2025.

### População e critérios de seleção

A população foi composta por estudantes da *Facultad de Enfermería N.º 2* da UAGro, do turno matutino, do segundo, terceiro e quarto anos. Convidou-se 460 estudantes a participar, dos quais 363 completaram o questionário, o que representa uma taxa de resposta de 78,9%. Foram incluídos estudantes que aceitaram participar de forma voluntária e registraram sua resposta por meio de um link individual enviado pelos representantes de turma via e-mail institucional. Foram excluídos os questionários incompletos e os registros duplicados.

### Instrumento de coleta de dados

O instrumento foi adaptado a partir de um questionário previamente validado e utilizado em outro estudo semelhante^17^. Realizaram-se adaptações na linguagem e no conteúdo para ajustá-lo ao contexto local dos estudantes de enfermagem da UAGro, mantendo a estrutura original das dimensões CAP. A validade de conteúdo foi revisada por duas docentes de enfermagem. Além da revisão por especialistas, realizou-se um estudo-piloto com um grupo de 12 estudantes que não fizeram parte da amostra final. Os estudantes verificaram a clareza, a compreensão e a pertinência dos itens, o que permitiu identificar e corrigir expressões ambíguas. Esse retorno assegurou que o questionário fosse compreensível para a população-alvo. A versão final do instrumento foi estruturada em quatro seções: características demográficas dos estudantes, que incluíram variáveis como idade, sexo, local de procedência, média acadêmica; conhecimento, avaliado por meio de 17 perguntas com opções de resposta “Sim” ou “Não”, atribuindo-se 1 ponto para “Sim” e 0 para “Não”. O conhecimento foi classificado como insatisfatório quando o estudante obteve ≤ 10 respostas corretas (≤ 60%) e satisfatório quando superou esse limiar; atitudes, que consistiram em 16 perguntas, avaliadas em uma escala Likert: 2 pontos para “Concordo”, 1 ponto para “Nem concordo nem discordo” e 0 pontos para “Discordo”. A atitude foi classificada como positiva quando a pontuação foi ≥ 60% e negativa quando foi menor; práticas, compostas por sete perguntas, que avaliaram a participação, a capacitação e a preparação dos estudantes na gestão de desastres. Cada resposta foi pontuada com 1 ponto para “Sim” e 0 para “Não”. Considerou-se prática satisfatória quando a pontuação foi ≥ 60% e insatisfatória quando ficou abaixo desse limiar.

Em comparação com o questionário original, nesta adaptação foram acrescentadas perguntas adicionais e modificada a distribuição de itens por dimensão, o que resultou em maior número de itens na dimensão de conhecimentos (17 vs. 16), em atitudes (16 vs. 11) e em práticas (7 vs. 3). Essas modificações foram realizadas com o propósito de ampliar a seção de práticas e adaptar o instrumento à realidade acadêmica e profissional dos estudantes de enfermagem em Acapulco, sem alterar a lógica da estrutura CAP. Além disso, foram integradas duas perguntas relativas à percepção da preparação institucional frente a desastres, com o objetivo de enriquecer a avaliação do contexto estudado.

### Validação e confiabilidade

A consistência interna do questionário foi avaliada por meio da fórmula Kuder-Richardson 20 (KR-20) para as seções com respostas dicotômicas. Os coeficientes de confiabilidade do KR-20 inferiores a 0,50 são considerados baixos; os compreendidos entre 0,50 e 0,80, moderados e superiores a 0,80, altos^22^. O alfa de Cronbach foi determinado para a seção de atitudes, sendo considerados aceitáveis os valores entre 0,75 e 0,90.

### Coleta dos dados

O convite foi gerenciado exclusivamente por meio dos representantes de turma, sem envolver o corpo docente, a fim de evitar vieses na relação professor-aluno. Inicialmente, o link foi enviado por e-mail institucional, juntamente com uma explicação sobre os objetivos e a relevância do estudo. Um segundo convite foi enviado 15 dias depois, com o intuito de aumentar a taxa de resposta. Cada estudante acessou o formulário mediante um registro único no Google *Forms*, o que garantiu a individualidade das respostas e evitou duplicidades.

### Tratamento e análise dos dados

Os dados foram organizados em uma planilha do *Microsoft Excel* e posteriormente analisados com o *software* estatístico R. Para as variáveis qualitativas (nominais e ordinais), foram calculadas frequências relativas e porcentagens, enquanto as variáveis contínuas foram expressas como média e desvio-padrão (DP). A comparação das variáveis categóricas foi realizada por meio do teste exato de Fisher. As correlações entre as pontuações de conhecimento, atitudes e práticas foram calculadas por meio do coeficiente de correlação de Pearson (r). Para cada correlação, foi obtido um intervalo de confiança de 95% (IC 95%). Considerou-se estatisticamente significativo um valor de p < 0,05.

### Aspectos éticos

O presente estudo atendeu aos princípios básicos da bioética e ao Código de Ética de Enfermagem, bem como ao disposto no Regulamento da Lei Geral de Saúde referente aos aspectos éticos da pesquisa em seres humanos. Além disso, foram respeitadas as convenções internacionais contidas na Declaração de Helsinki. O protocolo foi aprovado pelo Comitê de Ética em Pesquisa da *Facultad de Enfermería N.º 2* da UAGro, sob o número de protocolo PI-O1:10-11-2024.

## Resultados

A idade média dos estudantes que participaram do estudo foi de 21,54 anos (DP = 3,12). A maioria dos participantes encontrava-se no grupo etário de 21 a 23 anos (49,86%). Do total, 78,51% (285) eram mulheres, enquanto 21,49% (78) eram homens. Em relação à posição no curso, 43,25% (157) dos estudantes cursavam o quarto ano, 28,93% (105) o segundo ano e 27,82% (101) o terceiro ano. A maioria dos respondentes era solteira (95,04%), enquanto 3,03% eram casados e 1,93% viviam em união estável (Tabela 1). Em relação ao local de residência atual, 81,54% (296) residiam com a família e 18,46% (67) longe dela. A maioria dos estudantes era natural de Acapulco (79,06%), seguida por regiões como Costa Chica (7,99%) e Costa Grande (7,99%). A média do semestre anterior dos estudantes foi de 9,20 (DP = 0,49) (Tabela 1).

**Tabela 1:** Variáveis sociodemográficas e acadêmicas dos estudantes de enfermagem. Acapulco, Guerrero, México, 2024

Variável	Frequência (%)
Idade (anos)	
Média (DP*)	21,54 (3,12)
18-20	141 (38,84)
21-23	181 (49,86)
24 ou mais	41 (11,30)
Sexo	
Masculino	78 (21,49)
Feminino	285 (78,51)
Posição no curso	
Segundo	105 (28,93)
Terceiro	101 (27,82)
Quarto	157 (43,25)
Estado civil	
Solteiro	345 (95,04)
Casado	11 (3,03)
União estável	7 (1,93)
Com quem mora atualmente	
Com a família	296 (81,54)
Longe da família	67 (18,46)
Local de procedência	
Acapulco	287 (79,06)
Costa Chica	29 (7,99)
Costa Grande	29 (7,99)
Montaña	10 (2,75)
Tierra Caliente	3 (0,83)
Norte	1 (0,28)
Média do semestre anterior		
Média (DP*)	9,20 (0,49)

*DP = Desvio-padrão

A consistência interna da seção de conhecimento, avaliada por meio do coeficiente KR-20, foi de 0,61, o que indica confiabilidade moderada da escala. 

Entre os estudantes entrevistados, 97,25% (353) indicaram conhecer o conceito de desastre e 98,62% (358) reconheceram que a cidade onde residiam poderia sofrer um desastre em algum momento. Um total de 92,29% (335) dos estudantes indicou conhecer os desastres que podem afetar com maior probabilidade sua cidade e 97,80% (355) consideraram que um desastre poderia afetar as instalações de sua faculdade. Apenas 66,67% (242) receberam informações em sua formação acadêmica sobre a importância da organização para enfrentar desastres. Do total, 91,74% (333) dos estudantes afirmaram saber o que fazer em caso de um sismo ou terremoto; porém, apenas 65,01% (236) referiram estar familiarizados com os procedimentos de evacuação de emergência em sua faculdade. Além disso, 88,43% (321) dos estudantes indicaram saber quais ações tomar diante da chegada de um furacão ou tempestade tropical, mas somente 59,23% (215) afirmaram conhecer o lugar mais seguro durante uma inundação. O nível de conhecimento sobre preparação para desastres entre os estudantes de enfermagem foi predominantemente adequado.

De acordo com os resultados, 96,97% (352) dos participantes demonstraram nível adequado de conhecimento, enquanto apenas 3,03% (11) apresentaram nível inadequado. A média da pontuação foi de 14,54 (DP=2,07) (Tabela 2).

**Tabela 2:** Conhecimentos dos estudantes de enfermagem sobre a preparação para desastres. Acapulco, Guerrero, México, 2024

Perguntas sobre conhecimentos	Correto (%)	Incorreto (%)
1. Você já ouviu falar do conceito de desastre?	353 (97,25)	10 (2,75)
2. Durante sua formação na faculdade, falaram com você sobre a importância da organização para enfrentar desastres?	242 (66,67)	121 (33,33)
3. Os desastres são perturbações graves do funcionamento de uma comunidade que excedem sua capacidade de enfrentá-los com seus próprios recursos	320 (88,15)	43 (11,85)
4. São possíveis tanto desastres naturais quanto provocados pelo homem?	305 (84,02)	58 (15,98)
5. A cidade onde você reside atualmente poderia sofrer um desastre algum dia?	358 (98,62)	5 (1,38)
6. Você sabe quais são os desastres mais prováveis que podem afetar a cidade onde você reside?	335 (92,29)	28 (7,71)
7. Você acredita que um desastre poderia, em algum momento, afetar as instalações da sua faculdade de enfermagem?	355 (97,80)	8 (2,20)
8. O planejamento diante de desastres é o processo de preparação para as catástrofes, decidindo o que deve ser feito e como deve ser feito.	342 (94,21)	21 (5,79)
9. Você sabe por que é importante identificar e abordar as ameaças próximas que poderiam causar um desastre na sua faculdade ou comunidade?	333 (91,74)	30 (8,26)
10. Você está familiarizado com os procedimentos de evacuação de emergência na sua faculdade em caso de um desastre?	236 (65,01)	127 (34,99)
11. Você conhece as localizações exatas das portas de saída de emergência na sua faculdade?	245 (67,49)	118 (32,51)
12. Você sabe o que fazer em caso de um sismo ou terremoto?	333 (91,74)	30 (8,26)
13. Você sabe qual é o lugar mais seguro durante uma inundação?	215 (59,23)	148 (40,77)
14. Você sabe quais são as ações necessárias para se preparar diante da chegada de um furacão ou tempestade tropical?	321 (88,43)	42 (11,57)
15. Você sabe se, na sua cidade, as ações diante de desastres incluem a participação de pessoal de saúde (como médicos e enfermeiras) e de pessoal não sanitário (como bombeiros, policiais ou voluntários)?	291 (80,17)	72 (19,83)
16. Você sabe o que são as simulações?	358 (98,62)	5 (1,38)
17. Você compreende o papel que deve desempenhar durante uma simulação?	339 (93,39)	24 (6,61)
Nível de conhecimento	Adequado	352 (96,97)
	Inadequado	11 (3,03)
Pontuação média do nível de conhecimento	Média (DP*)	14,54 (2,07)

*DP = Desvio-padrão

A consistência interna da seção de atitudes, avaliada por meio do alfa de Cronbach, foi de 0,85, o que indica alta confiabilidade desta seção.

Do total, 91,46% (332) dos estudantes consideraram fundamental dispor de um plano de resposta diante de desastres e da necessidade de identificar e reduzir riscos potenciais. Além disso, 90,63% (329) declararam que a capacitação em preparação para desastres é essencial para todo o corpo universitário, enquanto 85,95% (312) referiram que essa capacitação deveria fazer parte integral da formação profissional dos estudantes de enfermagem. Um total de 85,67% (311) opinou que é importante que a universidade organize simulações de desastres de forma regular e 85,40% (310) informaram que esses exercícios melhorariam a preparação da comunidade universitária. Entre os respondentes, 66,39% (241) consideraram que a preparação para desastres deveria integrar o currículo obrigatório de todos os programas educacionais relacionados à área da saúde. Por outro lado, 88,15% (320) relataram que os estudantes de enfermagem deveriam ser capacitados não apenas na atenção em saúde, mas também na gestão emocional e psicológica durante desastres. Do total, 85,67% (311) assinalaram que é importante que os estudantes participem ativamente dos simulados e 80,72% (293) consideraram que esses exercícios deveriam incluir cenários de desastres naturais e emergências causadas por acidentes ou violência. Além disso, 90,36% (328) manifestaram concordar que os gestores universitários deveriam estar capacitados para liderar situações de desastre e 88,98% (323) reconheceram que a responsabilidade inclui todos os membros da comunidade universitária (Tabela 3).

O nível de atitude diante da preparação e resposta a desastres mostrou que 93,11% (338) dos estudantes apresentaram atitude positiva, enquanto 6,89% (25) apresentaram atitude negativa. A pontuação média de atitude foi de 26,79 (DP = 4,33), o que indica que, em geral, os estudantes demonstram disposição favorável em relação à preparação para desastres.

**Tabela 3:** Atitudes dos estudantes de enfermagem sobre a preparação para desastres. Acapulco, Guerrero, México, 2024

Perguntas sobre atitudes	Concordo (%)	Nem concordo nem discordo (%)	Discordo (%)
1. Não considero necessário receber informações sobre preparação e planejamento para desastres.	63 (17,36)	255 (70,25)	45 (12,40)
2. Você acredita que os gestores da universidade devem estar devidamente capacitados para liderar e enfrentar situações de desastre?	328 (90,36)	22 (6,06)	13 (3,58)
3. Você considera que a preparação e o planejamento diante de desastres são uma responsabilidade que inclui todos os membros da comunidade universitária (gestores, estudantes e pessoal administrativo)?	323 (88,98)	26 (7,16)	14 (3,86)
4. Considero importante identificar e reduzir os riscos potenciais que podem ocasionar desastres.	332 (91,46)	21 (5,79)	10 (2,75)
5. Considero fundamental dispor de um plano de resposta diante de desastres.	332 (91,46)	22 (6,06)	9 (2,48)
6. É importante que os planos de preparação diante de desastres sejam revisados e atualizados periodicamente para garantir sua efetividade.	327 (90,08)	26 (7,16)	10 (2,75)
7. Em que medida você concorda com a seguinte afirmação: “Considero que a probabilidade de ocorrer um desastre na minha faculdade é baixa”?	52 (14,33)	143 (39,39)	168 (46,28)
8. Em que medida você concorda com a seguinte afirmação: “É importante que a universidade organize simulados de desastres de forma regular para melhorar a preparação da comunidade”?	311 (85,67)	47 (12,95)	5 (1,38)
9. Considero que a capacitação em preparação para desastres é essencial para todo o pessoal universitário	329 (90,63)	29 (7,99)	5 (1,38)
10. Você considera fundamental que a capacitação em preparação para desastres faça parte integral da formação profissional dos estudantes de enfermagem?	312 (85,95)	42 (11,57)	9 (2,48)
11. Você considera que realizar simulações de desastres de forma regular na sua faculdade ajudaria a melhorar a preparação da comunidade universitária?	310 (85,40)	48 (13,22)	5 (1,38)
12. Os estudantes de enfermagem devem ser capacitados não apenas na atenção em saúde, mas também na gestão emocional e psicológica durante desastres.	320 (88,15)	37 (10,19)	6 (2,48)
13. Em que medida você concorda com a seguinte afirmação: “As simulações de desastres deveriam incluir tanto cenários de desastres naturais como emergências causadas por acidentes ou situações de violência”?	293 (80,72)	52 (14,33)	18 (4,96)
14. Em que medida você concorda com a seguinte afirmação: “É importante que os estudantes de enfermagem participem ativamente das simulações de desastres”?	311 (85,67)	46 (12,67)	6 (1,65)
15. Você considera que as simulações de desastre são uma boa oportunidade para melhorar a colaboração entre diferentes áreas da universidade.	298 (82,09)	57 (15,70)	8 (2,20)
16. A preparação diante de desastres deveria fazer parte do currículo obrigatório de todos os programas educacionais relacionados à área da saúde.	241 (66,39)	99 (27,27)	23 (6,34)
Nível de atitude	Positiva	338 (93,11)		
	Negativa	25 (6,89)		
Pontuação média de atitude	Média (DP*)	26,79 (4,33)		

*DP = Desvio-padrão

A consistência interna da seção de práticas, avaliada por meio do coeficiente KR-20, foi de 0,75, o que indica confiabilidade moderada da escala.

Na Tabela 4 são apresentados alguns dos resultados relacionados às práticas dos estudantes. Apenas 26,72% (97) dos estudantes receberam formação acadêmica continuada sobre gestão de desastres como parte de seu programa de estudos, enquanto 73,28% (266) não a receberam. Do total, 66,12% (240) dos respondentes mencionaram que em sua faculdade são realizadas simulações de desastres (sismos, inundações, incêndios, furacões), embora 33,88% (163) tenham indicado que eles não eram realizados. Somente 24,79% (90) compareceram a capacitações ou oficinas organizadas pela universidade sobre preparação para desastres. Um total de 22,87% (83) dos estudantes participou ativamente de atividades relacionadas à prevenção de desastres em sua comunidade universitária. Do total, 50,69% (184) dos estudantes referiram estar familiarizados com os protocolos de resposta diante de desastres naturais nos locais onde realizam suas práticas clínicas ou serviço social. Apenas 37,47% (136) receberam capacitação para realizar ações de primeiros socorros em caso de um desastre natural.

Somente 20,39% (74) dos estudantes apresentaram práticas adequadas, enquanto 79,61% (289) evidenciaram práticas inadequadas. A pontuação média de práticas foi de 2,85 (DP = 2,07), o que indica baixo nível de aplicação de medidas práticas em situações de emergência.

**Tabela 4:** Práticas dos estudantes de enfermagem sobre a preparação para desastres. Acapulco, Guerrero, México, 2024

Perguntas sobre práticas	Sim (%)	Não (%)
1. Você recebe formação acadêmica continuada sobre gestão de desastres como parte do seu programa de estudos?	97 (26,72)	266 (73,28)
2. Na sua faculdade, são realizadas simulações de desastres, como sismos, inundações, incêndios ou furacões?	240 (66,12)	123 (33,88)
3. Você já participou de capacitações ou oficinas sobre preparação para desastres organizadas pela sua universidade?	90 (24,79)	273 (75,21)
4. Você participa ativamente de atividades relacionadas à prevenção de desastres na sua comunidade universitária?	83 (22,87)	280 (77,13)
5. No local onde você realiza suas práticas clínicas, profissionais ou serviço social, você está familiarizado com os protocolos de resposta diante de desastres naturais?	184 (50,69)	179 (49,31)
6. Em caso de um desastre natural, você recebeu treinamento para realizar ações de primeiros socorros?	136 (37,47)	227 (62,53)
7. Em uma situação de desastre, você se sentiria capaz de coordenar ou apoiar os trabalhos de emergência na sua faculdade/instituição ou na sua cidade?	207 (57,02)	156 (42,98)
Nível de práticas	Adequadas	74 (20,39)
	Inadequadas	289 (79,61)
Pontuação média de práticas	Média (DP*)	2,85 (2,07)

*DP = Desvio-padrão

Quanto à percepção dos estudantes sobre a preparação institucional frente a desastres, apenas 20,66% (75) dos estudantes consideraram que os simulados realizados em sua universidade eram suficientes para prepará-los adequadamente diante de um desastre real, enquanto 48,21% (175) tiveram uma percepção neutra e 31,13% (113) opinaram que não são suficientes. Por outro lado, apenas 19,01% (69) dos estudantes perceberam que sua universidade estava bem preparada para enfrentar um desastre natural, enquanto 53,44% (194) manifestaram ter uma percepção neutra e 27,55% (100) consideraram que não estava.

Não foram encontradas diferenças significativas em conhecimento e atitude segundo idade, série, sexo, residência ou desempenho acadêmico (p > 0,05). No entanto, os estudantes do quarto ano realizaram mais práticas adequadas em comparação com os de anos inferiores (p = 0,03), enquanto idade, sexo, residência e desempenho acadêmico não influenciaram as práticas (p > 0,05) (Tabela 5).

**Tabela 5:** Distribuição do nível de conhecimento, atitude e práticas segundo características sociodemográficas dos estudantes. Acapulco, Guerrero, México, 2024

Idade (anos)				
	18-20 n (%)	21-23 n (%)	24 ou mais n (%)	p*
Conhecimento				
Inadequado	3 (0,83)	8 (2,20)	0 (0,00)	0,36
Adequado	138 (38,02)	173 (47,66)	41 (11,29)	
Atitude				
Positiva	132 (36,36)	167 (46,01)	39 (10,74)	0,83
Negativa	9 (2,48)	14 (3,86)	2 (0,65)	
Prática				
Adequada	118 (32,51)	141 (38,84)	30 (8,26)	0,23
Inadequada	23 (6,34)	40 (11,02)	11 (3,03)	
**Posição no curso (ano)**				
	**Segundo** **n (%)**	**Terceiro** **n (%)**	**Quarto** **n (%)**	
Conhecimento				
Inadequado	2 (0,55)	2 (0,55)	7 (1,93)	0,50
Adequado	103 (28,37)	99 (27,77)	150 (41,32)	
Atitude				
Positiva	99 (27,27)	96 (26,45)	143 (39,39)	0,46
Negativa	6 (1,65)	5 (1,38)	14 (3,86)	
Prática				
Adequada	13 (3,58)	21 (5,79)	40 (11,02)	0,03
Inadequada	92 (25,34)	80 (22,04)	117 (32,23)	
**Sexo**				
	**Masculino** **n (%)**		**Feminino** **n (%)**	
Conhecimento				
Inadequado	3 (0,83)		8 (2,20)	0,70
Adequado	75 (20,66)		277 (76,31)	
Atitude				
Positiva	69 (19,01)		269 (74,10)	0,07
Negativa	9 (2,48)		16 (4,41)	
Prática				
Adequada	22 (6,06)		52 (14,33)	0,06
Inadequada	56 (15,43)		233 (64,19)	
	**Com a família** **n (%)**		**Longe da família** **n (%)**	
Conhecimento				
Inadequado	11 (3,03)		0 (0,00)	0,22
Adequado	285 (78,51)		67 (18,46)	
Atitude				
Positiva	274 (75,48)		64 (17,63)	0,60
Negativa	22 (6,06)		3 (0,83)	
Prática				
Adequada	56 (15,43)		18 (4,96)	0,17
Inadequada	240 (66,12)		49 (13,50)	
**Média do semestre anterior**				
	**≥8,5** **n (%)**		**<8,5** **n (%)**	
Conhecimento				
Inadequado	10 (2,75)		1 (0,28)	1,00
Adequado	320 (88,15)		32 (8,82)	
Atitude				
Positiva	308 (84,85)		30 (8,26)	0,48
Negativa	22 (6,06)		3 (0,83)	
Prática				
Adequada	68 (18,73)		6 (1,65)	1,00
Inadequada	262 (72,18)		27 (7,44)	

*p = p-valor do teste exato de Fisher

Foram analisadas as pontuações totais de conhecimentos, atitudes e práticas por meio do coeficiente de correlação de Pearson. Observou-se correlação positiva moderada entre conhecimentos e práticas (r = 0,35; IC 95%: 0,25, 0,43), o que sugere que um nível mais elevado de conhecimento se associa a melhores práticas. A relação entre conhecimento e atitude foi fraca (r = 0,07; IC 95%: -0,03, 0,17), enquanto entre atitude e prática foi ligeiramente negativa (r = -0,07; IC 95%: -0,17, 0,02), sem significância estatística em ambos os casos.

## Discussão 

O aumento constante, a complexidade e a gravidade dos desastres em nível mundial e, em particular, no México, ressaltam a importância de dispor de pessoal de enfermagem capacitado que garanta uma atenção segura e oportuna em situações de emergência^23^. Este estudo avaliou pela primeira vez, na região sul do México, o nível de CAP em prevenção de desastres em estudantes de enfermagem da *Facultad de Enfermería N.º 2* de Acapulco, Guerrero, uma área altamente vulnerável a fenômenos naturais.

Os resultados mostram que a grande maioria dos estudantes (96,97%) apresentou nível adequado de conhecimento sobre preparação diante de desastres, valor superior ao relatado em Chipre e na Arábia Saudita^16^ ^-^ ^17^ e comparável ao de estudos multicêntricos na América Latina e no Caribe^10^. Isso sugere que, no contexto mexicano, os estudantes de enfermagem contam com uma base teórica sólida, provavelmente relacionada aos planos de estudo e à exposição a eventos catastróficos na região.

No que se refere às atitudes, 93,11% dos participantes manifestaram disposição positiva em relação à preparação e resposta diante de desastres, o que é coerente com achados internacionais que apontam atitudes favoráveis apesar de limitações em conhecimento técnico ou em experiência prática^11^ ^-^ ^12^ ^,^ ^18^. Estudos na Turquia demonstraram que cursos específicos em enfermagem de desastres melhoram a consciência e a autoeficácia dos estudantes, elementos estreitamente vinculados a uma atitude proativa diante de emergências^24^.

No entanto, as práticas adequadas foram limitadas: apenas 20,39% dos estudantes realizaram ações efetivas. Esse achado reflete uma lacuna crítica entre conhecimento e ação, padrão descrito em outros contextos nos quais, apesar de níveis adequados de conhecimento e de atitudes positivas, os estudantes carecem de experiência prática suficiente^16^ ^-^ ^19^. A exposição clínica progressiva parece exercer influência, uma vez que os estudantes do quarto ano realizaram significativamente mais práticas adequadas do que os de anos inferiores (p = 0,03).

A análise de correlação evidenciou relação moderada entre conhecimento e prática, enquanto a relação entre conhecimento e atitude, bem como entre atitude e prática, foi fraca e não significativa. Isso indica que a formação teórica e a motivação não se traduzem automaticamente em práticas efetivas, o que ressalta a necessidade de estratégias que integrem simulados, oficinas e experiências práticas para fortalecer a preparação efetiva dos estudantes.

O estudo aporta evidências relevantes sobre as discrepâncias entre os CAP dos estudantes de enfermagem no sul do México; complementa a literatura internacional e destaca a necessidade de incorporar competências práticas sistemáticas no plano de estudos. Atualmente, a graduação em enfermagem aborda temas de desastres em disciplinas isoladas, como saúde pública e primeiros socorros, porém não existe um curso integral que combine prevenção, mitigação e resposta diante de emergências. Dada a crescente frequência e intensidade de desastres vinculados às mudanças climáticas, torna-se prioritário reforçar a formação prática e a colaboração com organismos de proteção civil e de saúde.

Recomenda-se que as instituições de enfermagem implementem programas de formação continuada e oficinas práticas, especialmente em regiões com alta probabilidade de desastres^23^ ^-^ ^26^. Pesquisas futuras poderiam incorporar métodos qualitativos para explorar percepções, barreiras e necessidades dos estudantes, o que permitiria desenhar intervenções mais efetivas^15^ ^,^ ^26^.

Entre as limitações, destacam-se o desenho transversal, que impede estabelecer relações causais, e a inclusão de estudantes de uma única faculdade, o que pode limitar a generalização dos resultados. Sugere-se replicar o estudo com um desenho mais robusto e maior cobertura geográfica, a fim de fortalecer as conclusões.

## Conclusão

Os estudantes de enfermagem da *Facultad de Enfermería N.º 2* de Acapulco apresentaram nível adequado de conhecimentos e atitude positiva em relação à preparação diante de desastres. No entanto, as práticas adequadas foram limitadas, o que evidencia uma lacuna entre teoria e ação. Os estudantes do quarto ano mostraram melhores práticas do que os de anos inferiores, sugerindo a importância da formação progressiva e da experiência clínica. É necessário incorporar esses temas no plano de estudos e fortalecer a capacitação prática, uma vez que a maioria dos estudantes não estava familiarizada com os protocolos de resposta diante de desastres e com a gestão de primeiros socorros.

## Data Availability

Os conjuntos de dados relacionados a este artigo estarão disponíveis mediante solicitação ao autor correspondente.

# Translation: Conocimientos, actitudes y prácticas sobre preparación ante desastres en estudiantes de enfermería

**DOI:** 10.1590/1518-8345.8135.4854

**Authors:** Josefina Delgado-Delgado, Eduardo Pérez-Castro, Cornelio Bueno-Brito

**Affiliations:** 1 Universidad Autónoma de Guerrero, Facultad de Enfermería Nº 2, Acapulco, Guerrero, Mexico.; 2 Universidad Autónoma de Guerrero, Chilpancingo, Guerrero, Mexico.

**Keywords:** Enfermería, Estudiantes de Enfermería, Conocimiento, Actitudes, Prácticas, Preparación ante Desastres

## Abstract

**Objetivo:**

analizar los conocimientos, actitudes y prácticas sobre la preparación ante desastres en estudiantes de enfermería de un municipio altamente vulnerable a desastres naturales, en relación con su preparación ante estos eventos.

**Método:**

se aplicó una encuesta a 363 estudiantes, con una edad media de 21,54 años, en su mayoría mujeres (78,51%).

**Resultados:**

el 97,25% presentó conocimientos adecuados sobre desastres; no obstante, solo el 66,67% conocía los procedimientos de evacuación de su facultad. En cuanto a actitudes, el 93,11% mostró disposición positiva hacia la preparación ante desastres, aunque solo el 26,72% había recibido formación académica continua en el tema. Respecto a las prácticas, únicamente el 20,39% reportó acciones adecuadas en situaciones de emergencia y el 19,01% consideró que su universidad estaba preparada para enfrentar un desastre natural. Se identificó una correlación positiva entre el nivel de conocimiento y las prácticas, lo que sugiere que un mayor nivel de conocimiento favorece la aplicación de medidas efectivas en contextos de emergencia.

**Conclusión:**

aunque los estudiantes de enfermería mostraron conocimientos y actitudes favorables, las prácticas siguen siendo limitadas. Estos hallazgos evidencian la necesidad de reforzar la formación práctica en gestión de desastres para fortalecer su capacidad de respuesta ante emergencias.

## Destacados

**(1)** Primer estudio en México sobre preparación ante desastres en estudiantes de enfermería. **(2)** Conocimientos adecuados y actitudes favorables, pero prácticas limitadas en situaciones de desastres. **(3)** Un mayor nivel de conocimiento se asocia con mejores prácticas ante emergencias y desastres. **(4)** Solo el 26,72% recibió formación continua en gestión de desastres naturales.

## Introducción

De acuerdo con la Organización Mundial de la Salud, los desastres se definen como “situaciones imprevistas que representan serias e inmediatas amenazas para la salud pública o cualquier situación de salud pública que pone en peligro la vida o salud de una cantidad significativa de personas y exige acción inmediata”^1^. Los desastres suelen asociarse con graves crisis físicas, mentales, ambientales y económicas en la población afectada^2^ ^-^ ^4^. 

México es un país vulnerable a los desastres naturales^5^. En las últimas décadas, el país ha experimentado una gran variedad de desastres asociados a fenómenos naturales, principalmente huracanes, inundaciones y sismos^6^ ^-^ ^7^. En este contexto, Acapulco, un municipio situado en el estado de Guerrero, presenta una vulnerabilidad significativa debido a su ubicación geográfica y a su constante exposición a estos fenómenos. El 25 de octubre de 2023, Acapulco sufrió el devastador impacto del huracán Otis, de categoría 5, que ocasionó graves daños a la infraestructura, el medio ambiente y la población local^8^. Asimismo, las inundaciones ocasionadas por la tormenta tropical John a finales de septiembre de 2024 subrayaron la recurrencia y la magnitud de los desastres que afectan al municipio. Por ello, la prevención de desastres constituye una prioridad esencial para mitigar sus impactos negativos y proteger a las comunidades más vulnerables. 

Ante este panorama, diversos estudios han explorado los conocimientos, actitudes y prácticas (CAP) sobre la preparación ante desastres en estudiantes universitarios del área de la salud, quienes, como futuros profesionales sanitarios, deben estar adecuadamente preparados para desempeñar un papel clave en la gestión y la mitigación de los impactos de estos eventos^9^.

Un estudio multicéntrico realizado en 2.546 estudiantes de medicina de nueve países de América Latina y el Caribe tuvo por objetivo describir los conocimientos, percepciones y prácticas sobre riesgos y desastres. Los resultados revelaron que el 43,6% se consideró preparado para ayudar en caso de un desastre natural, mientras que el 91,2% consideró que los países de la región no estaban adecuadamente preparados para enfrentar este tipo de situaciones^10^. Por otro lado, en un estudio realizado en Líbano, los autores reportaron que los estudiantes de medicina presentaron un conocimiento moderado y una actitud favorable hacia la medicina de desastres, lo que subraya la necesidad de cursos específicos para fortalecer su preparación^11^. En Singapur, un estudio encontró que los estudiantes de medicina presentaron un nivel limitado de conocimientos sobre preparación para emergencias, además, la mayoría manifestó interés en incluir la medicina de desastres en el plan de estudios, con el fin de mejorar su capacidad de respuesta ante emergencias^12^.

En cuanto a los estudiantes de enfermería, diversos estudios han destacado la importancia de fortalecer su formación en la gestión de desastres, dado su papel clave en la atención primaria y en la respuesta inmediata ante emergencias^13^ ^-^ ^14^. Se ha identificado que estos estudiantes poseen una base de conocimientos, habilidades y capacidades que los convierten en activos valiosos y únicos durante los esfuerzos de respuesta ante desastres, lo que subraya la necesidad de mejorar su preparación para enfrentar este tipo de situaciones de forma eficaz^14^ ^-^ ^15^. Un estudio reciente en Chipre del Norte informó niveles moderados de preparación ante desastres en estudiantes de enfermería; y los autores destacaron la importancia de implementar una educación basada en el modelo de creencias en salud^16^. Una investigación realizada en 206 estudiantes de una facultad de enfermería en Arabia Saudita reveló que el 69% de los participantes tenían conocimientos adecuados, el 72% mostraba una actitud positiva hacia la preparación para desastres; sin embargo, el 84% presentaba una práctica inadecuada^17^. Un estudio identificó que la mayoría de los estudiantes mostró una actitud favorable hacia la preparación ante desastres y percibió una alta competencia en enfermería de desastres; sin embargo, presentó conocimientos insuficientes y una comprensión limitada de su rol en la respuesta ante emergencias^18^. Un estudio en Turquía halló que más de la mitad de los estudiantes de enfermería presentaba un nivel limitado de conocimientos sobre desastres, y destacó debilidades en la fase de rehabilitación, lo que subraya la importancia de fortalecer la formación práctica en gestión de emergencias^19^.

La formación de profesionales de enfermería constituye un pilar fundamental para la preparación y respuesta ante emergencias y desastres. Sin embargo, el plan de estudios de la Licenciatura en Enfermería de la Facultad de Enfermería N.º 2 de la Universidad Autónoma de Guerrero (UAGro) no contempla asignaturas específicas sobre gestión de desastres. Esta ausencia curricular adquiere especial relevancia en un municipio como Acapulco, uno de los más vulnerables a estos fenómenos naturales.

Actualmente, los desastres naturales son una preocupación creciente debido a su frecuencia e intensidad, así como su impacto en la salud pública, por lo que es necesario que los profesionales de la salud, especialmente las enfermeras y los enfermeros, estén preparados para responder de manera oportuna en estas situaciones críticas^20^. La evaluación de sus CAP en este ámbito permite identificar fortalezas y áreas de mejora en su formación, lo que garantiza que cuenten con las competencias necesarias para actuar de forma oportuna^14^ ^,^ ^20^. La adecuada preparación de los futuros profesionales de enfermería no solo permite una respuesta más eficiente ante los desastres, sino que también contribuye a la mitigación de sus impactos y a la protección de las poblaciones vulnerables. A partir de lo expuesto, el presente estudio tuvo como objetivo analizar los CAP de los estudiantes de Enfermería de un municipio altamente vulnerable a desastres naturales, en relación con su preparación ante estos eventos.

## Método

### Diseño del estudio 

Se realizó un estudio observacional de corte transversal a fin de explorar los CAP de los estudiantes de enfermería en relación con la prevención de desastres, siguiendo las recomendaciones de la guía *Strengthening the Reporting of Observational Studies in Epidemiology* (STROBE) para estudios observacionales^21^.

### Lugar

El estudio se llevó a cabo en la Facultad de Enfermería N.º 2 de la Universidad Autónoma de Guerrero (UAGro), ubicada en la ciudad y puerto de Acapulco, Guerrero, México.

### Período

La recolección de datos se realizó entre el 25 de noviembre de 2024 y el 20 de enero de 2025.

### Población y criterios de selección

La población estuvo compuesta por estudiantes de la Facultad de Enfermería N.º 2 de la UAGro, en el turno matutino, de segundo, tercero y cuarto año. Se invitó a 460 estudiantes a participar, de los cuales 363 completaron el cuestionario, lo que representa una tasa de respuesta del 78,9%. Se incluyeron estudiantes que aceptaron participar de forma voluntaria y registraron su respuesta mediante un enlace individual enviado por los jefes de grupo a través del correo institucional. Se excluyeron los cuestionarios incompletos y los registros duplicados.

### Instrumento de recolección de datos

El instrumento fue adaptado a partir de un cuestionario previamente validado y empleado en otro estudio similar^17^. Se realizaron adaptaciones en el lenguaje y en el contenido para ajustarlo al contexto local de los estudiantes de enfermería de la UAGro, manteniendo la estructura original de las dimensiones CAP. La validez de contenido fue revisada por dos docentes de enfermería. Además de la revisión por expertos, se realizó una prueba piloto con un grupo de 12 estudiantes que no formaron parte de la muestra final. Los estudiantes verificaron la claridad, comprensión y pertinencia de los ítems, lo que permitió identificar y corregir expresiones ambiguas. Esta retroalimentación aseguró que el cuestionario fuera comprensible para la población objetivo. La versión final del instrumento estuvo estructurada en cuatro secciones: características demográficas de los estudiantes, que incluyeron variables como edad, sexo, lugar de procedencia, promedio académico; conocimiento, evaluado a través de 17 preguntas con opciones de respuesta “Sí” o “No”, asignando 1 punto a “Sí” y 0 a “No”. Se clasificó el conocimiento como insatisfactorio si el estudiante obtuvo ≤ 10 respuestas correctas (≤ 60%) y satisfactorio si superó este umbral; actitudes, que consistieron en 16 preguntas, evaluadas en una escala de Likert: 2 puntos para “De acuerdo”, 1 punto para “Ni de acuerdo ni en desacuerdo” y 0 puntos para “En desacuerdo”. La actitud se clasificó como positiva si la puntuación era ≥ 60% y negativa si era menor; prácticas, conformadas por siete preguntas, que evaluaron la participación, capacitación y preparación de los estudiantes en la gestión de desastres. Cada respuesta se calificó con 1 punto para “Sí” y 0 para “No”. Se consideró práctica satisfactoria con una puntuación ≥60% e insatisfactoria por debajo de este umbral.

En comparación con el cuestionario original, en esta adaptación se añadieron preguntas adicionales y se modificó la distribución de ítems por dimensión, lo que se tradujo en un mayor número de ítems en la dimensión de conocimientos (17 vs. 16), en actitudes (16 vs. 11) y en prácticas (7 vs. 3). Estas modificaciones se realizaron con el propósito de ampliar la sección de prácticas y adaptar el instrumento a la realidad académica y profesional de los estudiantes de enfermería en Acapulco, sin alterar la lógica de la estructura CAP. Además, se integraron dos preguntas relativas a la percepción de la preparación institucional frente a desastres, con el fin de enriquecer la evaluación del contexto estudiado.

### Validación y confiabilidad

La consistencia interna del cuestionario se evaluó mediante la fórmula Kuder-Richardson 20 (KR-20) para las secciones con respuestas dicotómicas. Los coeficientes de fiabilidad del KR-20 inferiores a 0,50 se consideran bajos; los comprendidos entre 0,50 y 0,80, moderados y superiores a 0,80, altos^22^. El alfa de Cronbach se determinó para la sección de actitudes, considerándose aceptables los valores entre 0,75 y 0,90.

### Recolección de los datos

La invitación se gestionó exclusivamente a través de los jefes de grupo, sin involucrar al cuerpo docente, a fin de evitar sesgos en la relación profesor-alumno. Inicialmente, se envió el enlace mediante correo institucional, junto con una explicación de los objetivos y de la relevancia del estudio. Se envió una segunda invitación 15 días después, con el fin de incrementar la tasa de respuesta. Cada estudiante accedió mediante un registro único en *Google Forms*, lo que garantizó la individualidad de las respuestas y evitó duplicados.

### Tratamiento y análisis de los datos

Los datos se organizaron en una hoja de cálculo de *Microsoft Excel* y posteriormente se analizaron con el *software* estadístico R. Para las variables cualitativas (nominales y ordinales), se calcularon frecuencias relativas y porcentajes, mientras que las variables continuas se expresaron como media y desviación estándar (DE). La comparación de variables categóricas se realizó mediante la prueba exacta de Fisher. Se calcularon las correlaciones entre las puntuaciones de conocimiento, actitudes y prácticas mediante el coeficiente de correlación de Pearson (r). Para cada correlación, se obtuvo un intervalo de confianza del 95% (IC 95%). Se consideró estadísticamente significativo un valor de p < 0,05.

### Aspectos éticos

El presente estudio se apegó a los principios básicos de bioética y al Código de Ética de Enfermería, así como a lo establecido en el Reglamento de la Ley General de Salud respecto de los aspectos éticos de la investigación en seres humanos. Además, se respetaron las convenciones internacionales contenidas en la Declaración de Helsinki. El protocolo fue aprobado por el Comité de Ética en Investigación de la Facultad de Enfermería N.º 2 de la UAGro, con el folio PI-O1:10-11-2024.

## Resultados

La edad media de los estudiantes que participaron en el estudio fue de 21,54 años (DE = 3,12). La mayoría de los participantes se encontraba dentro del grupo de edad de 21 a 23 años (49,86%). El 78,51% (285) fueron mujeres, mientras que el 21,49% (78) fueron hombres. Respecto del grado escolar, el 43,25% (157) de los estudiantes cursaban el cuarto año, el 28,93% (105) el segundo año y el 27,82% (101) el tercer año. La mayoría de los encuestados eran solteros (95,04%), mientras que un 3,03% estaban casados y el 1,93% vivían en unión libre (Tabla 1). En relación con su lugar de residencia actual, el 81,54% (296) vivía con su familia y el 18,46% (67) lejos de ella. La mayoría de los estudiantes eran originarios de Acapulco (79,06%), seguidos por regiones como Costa Chica (7,99%) y Costa Grande (7,99%). El promedio del semestre anterior de los estudiantes fue de 9,20 (DE = 0,49) (Tabla 1).

**Tabla 1:** Variables sociodemográficas y académicas de los estudiantes de enfermería. Acapulco, Guerrero, México, 2024

Variable	Frecuencia (%)
Edad (años)	
Media (DE*)	21,54 (3,12)
18-20	141 (38,84)
21-23	181 (49,86)
24 o más	41 (11,30)
Sexo	
Masculino	78 (21,49)
Femenino	285 (78,51)
Grado escolar	
Segundo	105 (28,93)
Tercero	101 (27,82)
Cuarto	157 (43,25)
Estado civil	
Soltero	345 (95,04)
Casado	11 (3,03)
Unión libre	7 (1,93)
Con quien vive actualmente	
Con su familia	296 (81,54)
Lejos de su familia	67 (18,46)
Lugar de procedencia	
Acapulco	287 (79,06)
Costa Chica	29 (7,99)
Costa Grande	29 (7,99)
Montaña	10 (2,75)
Tierra Caliente	3 (0,83)
Norte	1 (0,28)
Promedio del semestre anterior		
Media (DE*)	9,20 (0,49)

*DE = Desviación Estándar

La consistencia interna de la sección de conocimiento evaluada mediante el coeficiente KR-20 fue de 0,61, lo que indica una fiabilidad moderada de la escala. 

El 97,25% (353) de los estudiantes encuestados indicó conocer el concepto de desastre, y el 98,62% (358) reconocieron que la ciudad donde residían podría sufrir un desastre en algún momento. Un 92,29% (335) de los estudiantes indicaron conocer los desastres que pueden afectar con mayor probabilidad su ciudad, y el 97,80% (355) consideró que un desastre podría afectar las instalaciones de su facultad. Solo el 66,67% (242) recibió información en su formación académica sobre la importancia de la organización para enfrentar desastres. El 91,74% (333) de los estudiantes afirmaron saber qué hacer en caso de un sismo o terremoto, solo el 65,01% (236) refirieron estar familiarizados con los procedimientos de evacuación de emergencia en su facultad. El 88,43% (321) de los estudiantes indicó saber qué acciones tomar ante la llegada de un huracán o tormenta tropical, pero únicamente el 59,23% (215) afirmó conocer el lugar más seguro durante una inundación. El nivel de conocimiento sobre preparación ante desastres entre los estudiantes de enfermería fue predominantemente adecuado.

De acuerdo con los resultados, el 96,97% (352) de los participantes demostraron un nivel de conocimiento adecuado, mientras que solo el 3,03% (11) presentaron un nivel inadecuado. El promedio de la puntuación fue de 14,54 (DE=2,07) (Tabla 2).

**Tabla 2:** Conocimientos de los estudiantes de enfermería sobre la preparación ante desastres. Acapulco, Guerrero, México, 2024

Preguntas sobre conocimientos	Correcto (%)	Incorrecto (%)
1. ¿Has oído hablar del concepto de desastre?	353 (97,25)	10 (2,75)
2. ¿Durante tu formación en la facultad te han hablado sobre la importancia de estar organizados para enfrentar desastres?	242 (66,67)	121 (33,33)
3. Los desastres son perturbaciones graves del funcionamiento de una comunidad que exceden su capacidad para hacer frente con sus propios recursos	320 (88,15)	43 (11,85)
4. ¿Son posibles tanto desastres naturales como provocados por el hombre?	305 (84,02)	58 (15,98)
5. ¿La ciudad donde radicas actualmente podría sufrir un desastre algún día?	358 (98,62)	5 (1,38)
6. ¿Sabes cuáles son los desastres más probables que pueden afectar a la ciudad donde resides?	335 (92,29)	28 (7,71)
7. ¿Crees que un desastre podría afectar en algún momento las instalaciones de tu facultad de enfermería?	355 (97,80)	8 (2,20)
8. La planificación ante desastres es el proceso de preparación para las catástrofes decidiendo qué debe hacerse y cómo debe hacerse.	342 (94,21)	21 (5,79)
9. ¿Sabes por qué es importante identificar y abordar las amenazas cercanas que podrían causar un desastre en tu facultad o comunidad?	333 (91,74)	30 (8,26)
10. ¿Estás familiarizado con los procedimientos de evacuación de emergencia en tu facultad en caso de un desastre?	236 (65,01)	127 (34,99)
11. ¿Conoces las ubicaciones exactas de las puertas de salida de emergencia en tu facultad?	245 (67,49)	118 (32,51)
12. ¿Sabes qué hacer en caso de un sismo o terremoto?	333 (91,74)	30 (8,26)
13. ¿Sabes cuál es el lugar más seguro durante una inundación?	215 (59,23)	148 (40,77)
14. ¿Sabes cuáles son las acciones necesarias para prepararte ante la llegada de un huracán o tormenta tropical?	321 (88,43)	42 (11,57)
15. ¿Sabes si en tu ciudad las acciones ante desastres incluyen la participación de personal de salud (como médicos y enfermeras) y del personal no sanitario (como bomberos, policías o voluntarios)?	291 (80,17)	72 (19,83)
16. ¿Sabes qué son los simulacros?	358 (98,62)	5 (1,38)
17. ¿Comprendes el papel que debes desempeñar durante un simulacro?	339 (93,39)	24 (6,61)
Nivel de conocimiento	Adecuado	352 (96,97)
	Inadecuado	11 (3,03)
Puntuación media del nivel de conocimiento	Media (DE*)	14,54 (2,07)

*DE = Desviación Estándar

La consistencia interna de la sección de actitudes evaluada mediante el alfa de Cronbach fue de 0,85, lo que indica una alta fiabilidad de esta sección. 

El 91,46% (332) de los estudiantes consideró fundamental contar con un plan de respuesta ante desastres y la necesidad de identificar y reducir riesgos potenciales. El 90,63% (329) declaró que la capacitación en preparación para desastres es esencial para todo el personal universitario, mientras que el 85,95% (312) refirió que esta capacitación debería formar parte integral de la formación profesional de los estudiantes de enfermería. El 85,67% (311) opinó que es importante que la universidad organice simulacros de desastres regularmente, y el 85,40% (310) informó que estos ejercicios mejorarían la preparación de la comunidad universitaria. El 66,39% (241) de los encuestados consideró que la preparación ante desastres debería ser parte del currículo obligatorio en todos los programas educativos relacionados con el área de la salud. Por otro lado, el 88,15% (320) reportó que los estudiantes de enfermería deberían estar capacitados no solo en la atención médica, sino también en la gestión emocional y psicológica durante desastres. El 85,67% (311) señaló que es importante que los estudiantes participen activamente en simulacros, y el 80,72% (293) consideró que estos ejercicios deberían incluir escenarios de desastres naturales y emergencias causadas por accidentes o violencia. El 90,36% (328) manifestó estar de acuerdo en que los directivos universitarios debían estar capacitados para liderar situaciones de desastre, y el 88,98% (323) reconoció que la responsabilidad incluye a todos los miembros de la comunidad universitaria (Tabla 3).

El nivel de actitud ante a la preparación y respuesta ante desastres mostró que el 93,11% (338) de los estudiantes presentaron una actitud positiva, mientras que el 6,89% (25) presentaron una actitud negativa. La puntuación media de actitud fue de 26,79 (DE= 4,33), lo que indica que, en general, los estudiantes muestran una disposición favorable hacia la preparación ante desastres.

**Tabla 3:** Actitudes de los estudiantes de enfermería sobre la preparación ante desastres. Acapulco, Guerrero, México, 2024

Preguntas sobre actitudes	De acuerdo (%)	Ni de acuerdo ni en desacuerdo (%)	En desacuerdo (%)
1. No considero necesario recibir información sobre preparación y planificación ante desastres.	63 (17,36)	255 (70,25)	45 (12,40)
2. ¿Crees que los directivos de la universidad deben estar debidamente capacitados para liderar y enfrentar situaciones de desastre?	328 (90,36)	22 (6,06)	13 (3,58)
3. ¿Consideras que la preparación y planificación ante desastres es una responsabilidad que incluye a todos los miembros de la comunidad universitaria (directivos, estudiantes y personal administrativo)?	323 (88,98)	26 (7,16)	14 (3,86)
4. Considero que es importante identificar y reducir los riesgos potenciales que pueden ocasionar desastres	332 (91,46)	21 (5,79)	10 (2,75)
5. Considero que es fundamental contar con un plan de respuesta ante desastres.	332 (91,46)	22 (6,06)	9 (2,48)
6. Es importante que los planes de preparación ante desastres sean revisados y actualizados periódicamente para garantizar su efectividad.	327 (90,08)	26 (7,16)	10 (2,75)
7. ¿Qué tan de acuerdo estás con la siguiente afirmación: “Considero que la probabilidad de que ocurra un desastre en mi facultad es baja”?	52 (14,33)	143 (39,39)	168 (46,28)
8. ¿Qué tan de acuerdo estás con la siguiente afirmación: “Es importante que la universidad organice simulacros de desastres de manera regular para mejorar la preparación de la comunidad”?	311 (85,67)	47 (12,95)	5 (1,38)
9. Considero que la capacitación en preparación para desastres es esencial para todo el personal universitario	329 (90,63)	29 (7,99)	5 (1,38)
10. ¿Consideras fundamental que la capacitación en preparación para desastres forme parte integral de la formación profesional de los estudiantes de enfermería?	312 (85,95)	42 (11,57)	9 (2,48)
11. ¿Consideras que realizar simulacros de desastres de manera regular en tu facultad ayudaría a mejorar la preparación de la comunidad universitaria?	310 (85,40)	48 (13,22)	5 (1,38)
12. Los estudiantes de enfermería deben estar capacitados no solo en la atención médica, sino también en la gestión emocional y psicológica durante desastres.	320 (88,15)	37 (10,19)	6 (2,48)
13. ¿Qué tan de acuerdo estás con la siguiente afirmación: “Los simulacros de desastres deberían incluir tanto escenarios de desastres naturales como emergencias causadas por accidentes o situaciones de violencia”?	293 (80,72)	52 (14,33)	18 (4,96)
14. ¿Qué tan de acuerdo estás con la siguiente afirmación: “Es importante que los estudiantes de enfermería participen activamente en los simulacros de desastres”?	311 (85,67)	46 (12,67)	6 (1,65)
15. Consideras que los simulacros de desastre son una buena oportunidad para mejorar la colaboración entre diferentes áreas de la universidad.	298 (82,09)	57 (15,70)	8 (2,20)
16. La preparación ante desastres debería ser parte del currículo obligatorio de todos los programas educativos relacionados del área de la salud.	241 (66,39)	99 (27,27)	23 (6,34)
Nivel de actitud	Positiva	338 (93,11)		
	Negativa	25 (6,89)		
Puntuación media de actitud	Media (DE*)	26,79 (4,33)		

*DE = Desviación Estándar

La consistencia interna de la sección de prácticas evaluada mediante el coeficiente KR-20 fue de 0,75, lo que indica una fiabilidad moderada de la escala. 

En la Tabla 4 se presentan algunos de los resultados relacionados con las prácticas de los estudiantes. Solo el 26,72% (97) de los estudiantes recibieron formación académica continua sobre gestión de desastres como parte de su programa de estudios, mientras que el 73,28% (266) no la recibieron. El 66,12% (240) de los encuestados mencionaron que en su escuela se realizan simulacros de desastres (sismos, inundaciones, incendios, huracanes), aunque un 33,88% (163) indicó que no se llevaban a cabo. Solo el 24,79% (90) asistieron a capacitaciones o talleres organizados por la universidad sobre preparación ante desastres. El 22,87% (83) de los estudiantes participaron activamente en actividades relacionadas con la prevención de desastres en su comunidad universitaria. El 50,69% (184) de los estudiantes refirieron estar familiarizados con los protocolos de respuesta ante desastres naturales en los lugares donde realizan sus prácticas clínicas o servicio social. Solo el 37,47% (136) recibió capacitación para realizar acciones de primeros auxilios en caso de un desastre natural.

Solo el 20,39% (74) de los estudiantes presentaron prácticas adecuadas, mientras que el 79,61% (289) reflejaron prácticas inadecuadas. La puntuación media de prácticas fue de 2,85 (DE = 2,07), lo que indica un bajo nivel de aplicación de medidas prácticas en situaciones de emergencia.

**Tabla 4:** Prácticas de los estudiantes de enfermería sobre la preparación ante desastres. Acapulco, Guerrero, México, 2024

Preguntas sobre prácticas	Sí (%)	No (%)
1. ¿Recibes formación académica continua sobre gestión de desastres como parte de tu programa de estudios?	97 (26,72)	266 (73,28)
2. En tu escuela, ¿se realizan simulacros de desastres, como sismos, inundaciones, incendios o huracanes?	240 (66,12)	123 (33,88)
3. ¿Has asistido a capacitaciones o talleres sobre preparación ante desastres organizados por tu universidad?	90 (24,79)	273 (75,21)
4. ¿Participas activamente en actividades relacionadas con la prevención de desastres en tu comunidad universitaria?	83 (22,87)	280 (77,13)
5. En el lugar donde realizas tus prácticas clínicas, profesionales o servicio social, ¿estás familiarizado con los protocolos de respuesta ante desastres naturales?	184 (50,69)	179 (49,31)
6. En caso de un desastre natural, ¿has recibido entrenamiento para realizar acciones de primeros auxilios?	136 (37,47)	227 (62,53)
7. En una situación de desastre, ¿te sentirías capaz de coordinar o apoyar las labores de emergencia en tu facultad/institución o en tu ciudad?	207 (57,02)	156 (42,98)
Nivel de prácticas	Adecuadas	74 (20,39)
	Inadecuadas	289 (79,61)
Puntuación media de prácticas	Media (DE*)	2,85 (2,07)

*DE = Desviación Estándar

Respecto de la percepción de los estudiantes sobre la preparación institucional frente a desastres, solo el 20,66% (75) de los estudiantes consideraron que los simulacros realizados en su universidad eran suficientes para prepararlos adecuadamente ante un desastre real, mientras que el 48,21% (175) tuvo una percepción neutra y un 31,13% (113) opinó que no son suficientes. Por otro lado, solo el 19,01% (69) de los estudiantes percibieron que su universidad estaba bien preparada para afrontar un desastre natural, mientras que el 53,44% (194) manifestó tener una percepción neutra y el 27,55% (100) consideró que no lo estaba.

No se encontraron diferencias significativas en conocimiento y actitud según edad, grado escolar, sexo, residencia o rendimiento académico (p > 0,05). Sin embargo, los estudiantes de cuarto año realizaron más prácticas adecuadas en comparación con los de años inferiores (p = 0,03), mientras que edad, sexo, residencia y rendimiento académico no influyeron en las prácticas (p > 0,05) (Tabla 5).

**Tabla 5:** Distribución del nivel de conocimiento, actitud y prácticas según características sociodemográficas de los estudiantes. Acapulco, Guerrero, México, 2024

Edad (años)				
	18-20 n (%)	21-23 n (%)	24 o más n (%)	p*
Conocimiento				
Inadecuado	3 (0,83)	8 (2,20)	0 (0,00)	0,36
Adecuado	138 (38,02)	173 (47,66)	41 (11,29)	
Actitud				
Positiva	132 (36,36)	167 (46,01)	39 (10,74)	0,83
Negativa	9 (2,48)	14 (3,86)	2 (0,65)	
Práctica				
Adecuada	118 (32,51)	141 (38,84)	30 (8,26)	0,23
Inadecuada	23 (6,34)	40 (11,02)	11 (3,03)	
**Grado escolar**				
	**Segundo** **n (%)**	**Tercero** **n (%)**	**Cuarto** **n (%)**	
Conocimiento				
Inadecuado	2 (0,55)	2 (0,55)	7 (1,93)	0,50
Adecuado	103 (28,37)	99 (27,77)	150 (41,32)	
Actitud				
Positiva	99 (27,27)	96 (26,45)	143 (39,39)	0,46
Negativa	6 (1,65)	5 (1,38)	14 (3,86)	
Práctica				
Adecuada	13 (3,58)	21 (5,79)	40 (11,02)	0,03
Inadecuada	92 (25,34)	80 (22,04)	117 (32,23)	
**Sexo**				
	**Masculino n (%)**		**Femenino n (%)**	
Conocimiento				
Inadecuado	3 (0,83)		8 (2,20)	0,70
Adecuado	75 (20,66)		277 (76,31)	
Actitud				
Positiva	69 (19,01)		269 (74,10)	0,07
Negativa	9 (2,48)		16 (4,41)	
Práctica				
Adecuada	22 (6,06)		52 (14,33)	0,06
Inadecuada	56 (15,43)		233 (64,19)	
	**Con su familia** **n (%)**		**Lejos de su familia** **n (%)**	
Conocimiento				
Inadecuado	11 (3,03)		0 (0,00)	0,22
Adecuado	285 (78,51)		67 (18,46)	
Actitud				
Positiva	274 (75,48)		64 (17,63)	0,60
Negativa	22 (6,06)		3 (0,83)	
Práctica				
Adecuada	56 (15,43)		18 (4,96)	0,17
Inadecuada	240 (66,12)		49 (13,50)	
**Promedio del semestre anterior**				
	**≥8,5** **n (%)**		**<8,5** **n (%)**	
Conocimiento				
Inadecuado	10 (2,75)		1 (0,28)	1,00
Adecuado	320 (88,15)		32 (8,82)	
Actitud				
Positiva	308 (84,85)		30 (8,26)	0,48
Negativa	22 (6,06)		3 (0,83)	
Práctica				
Adecuada	68 (18,73)		6 (1,65)	1,00
Inadecuada	262 (72,18)		27 (7,44)	

*p = p-valor de la prueba Exacta de Fisher

Se analizaron las puntuaciones totales de conocimientos, actitudes y prácticas mediante el coeficiente de correlación de Pearson. Se observó una correlación positiva moderada entre conocimientos y prácticas (r = 0,35; IC 95%: 0,25, 0,43), lo que sugiere que un mayor nivel de conocimiento se asocia con mejores prácticas. La relación entre conocimiento y actitud fue débil (r = 0,07; IC 95%: -0,03, 0,17), mientras que entre actitud y práctica fue ligeramente negativa (r = -0,07; IC 95%: -0,17, 0,02), sin significancia estadística en ambos casos.

## Discusión 

El aumento constante, la complejidad y la gravedad de los desastres a nivel mundial y, en particular, en México, subrayan la importancia de disponer de personal de enfermería capacitado que garantice una atención segura y oportuna en situaciones de emergencia^23^. Este estudio evaluó, por primera vez en la región sur de México, el nivel de CAP en prevención de desastres en estudiantes de enfermería de la Facultad de Enfermería N.º 2 de Acapulco, Guerrero, una zona altamente vulnerable a fenómenos naturales.

Los resultados muestran que la gran mayoría de los estudiantes (96,97%) mostró un nivel adecuado de conocimiento sobre preparación ante desastres, cifra superior a la reportada en Chipre y Arabia Saudita^16^ ^-^ ^17^ y comparable con estudios multicéntricos en América Latina y el Caribe^10^. Esto sugiere que, en el contexto mexicano, los estudiantes de enfermería cuentan con una base teórica sólida, probablemente relacionada con los planes de estudio y la exposición a eventos catastróficos en la región.

En cuanto a las actitudes, el 93,11% de los participantes manifestaron disposición positiva hacia la preparación y respuesta ante desastres, lo cual es coherente con hallazgos internacionales que muestran actitudes favorables a pesar de limitaciones en conocimiento técnico o en la experiencia práctica^11^ ^-^ ^12^ ^,^ ^18^. Estudios en Turquía han demostrado que los cursos específicos en enfermería de desastres mejoran la conciencia y la autoeficacia de los estudiantes, elementos estrechamente vinculados con la actitud proactiva ante emergencias^24^.

No obstante, las prácticas adecuadas fueron limitadas, solo el 20,39% de los estudiantes realizaron acciones efectivas. Este hallazgo refleja una brecha crítica entre los conocimientos y la acción, un patrón descrito en otros contextos en los que, a pesar de niveles adecuados de conocimiento y actitudes positivas, los estudiantes carecen de experiencia práctica suficiente^16^ ^-^ ^19^. La exposición clínica progresiva parece influir, ya que los estudiantes de cuarto año realizaron significativamente más prácticas adecuadas que los de años inferiores (p = 0,03).

El análisis de correlación evidenció una relación moderada entre conocimiento y práctica, mientras que la relación entre conocimiento y actitud, así como entre actitud y práctica, fue débil y no significativa. Esto indica que la formación teórica y la motivación no se traducen automáticamente en prácticas efectivas, lo que subraya la necesidad de estrategias que integren simulacros, talleres y experiencias prácticas para fortalecer la preparación efectiva de los estudiantes.

El estudio aporta evidencia relevante sobre las discrepancias entre los CAP de los estudiantes de enfermería en el sur de México; complementa la literatura internacional y resalta la necesidad de incorporar competencias prácticas sistemáticas en el plan de estudios. Actualmente, la licenciatura en enfermería aborda temas de desastres en asignaturas aisladas, como salud pública y primeros auxilios, pero no existe un curso integral que combine prevención, mitigación y respuesta ante emergencias. Dada la creciente frecuencia e intensidad de desastres vinculados al cambio climático, resulta prioritario reforzar la formación práctica y la colaboración con organismos de protección civil y de salud.

Se recomienda que las instituciones de enfermería implementen programas de formación continua y talleres prácticos, especialmente en regiones con alta probabilidad de desastres^23^ ^-^ ^26^. Futuras investigaciones podrían incorporar métodos cualitativos para explorar percepciones, barreras y necesidades de los estudiantes, lo que permitiría diseñar intervenciones más efectivas^15^ ^,^ ^26^.

Entre las limitaciones, destacan el diseño de corte transversal, que impide establecer relaciones causales, y la inclusión de estudiantes de una sola facultad, lo que puede limitar la generalización de los resultados. Se sugiere replicar el estudio con un diseño más robusto y una mayor cobertura geográfica, a fin de fortalecer las conclusiones.

## Conclusión

Los estudiantes de enfermería de la Facultad de Enfermería N.º 2 de Acapulco presentaron un nivel adecuado de conocimientos y una actitud positiva hacia la preparación ante desastres. Sin embargo, las prácticas adecuadas fueron limitadas, lo que evidencia una brecha entre teoría y acción. Los estudiantes de cuarto año mostraron mejores prácticas que los de años inferiores, lo que sugiere la importancia de la formación progresiva y la experiencia clínica. Es necesario incorporar estos temas en el plan de estudios y fortalecer la capacitación práctica, ya que la mayoría de los estudiantes no estaban familiarizados con los protocolos de respuesta ante desastres y la gestión de primeros auxilios.

## Data Availability

Los conjuntos de datos relacionados con este artículo estarán disponibles previa solicitud al autor correspondiente.
